# Modelling evolution of virulence in populations with a distributed parasite load

**DOI:** 10.1007/s00285-019-01351-6

**Published:** 2019-04-10

**Authors:** Simran K. Sandhu, Andrew Yu. Morozov, József Z. Farkas

**Affiliations:** 1 Department of Mathematics, University of Leicester, Leicester, LE1 7RH UK; 2 Division of Computing Science and Mathematics, University of Stirling, Stirling, FK9 4LA UK

**Keywords:** Structured populations, Infection load, Evolutionary attractor, Pairwise invasibility plot (PIP), Singular points, Trade-off, Stability

## Abstract

Modelling evolution of virulence in host-parasite systems is an actively developing area of research with ever-growing literature. However, most of the existing studies overlook the fact that individuals within an infected population may have a variable infection load, i.e. infected populations are naturally structured with respect to the parasite burden. Empirical data suggests that the mortality and infectiousness of individuals can strongly depend on their infection load; moreover, the shape of distribution of infection load may vary on ecological and evolutionary time scales. Here we show that distributed infection load may have important consequences for the eventual evolution of virulence as compared to a similar model without structuring. Mathematically, we consider an SI model, where the dynamics of the infected subpopulation is described by a von Förster-type equation, in which the infection load plays the role of age. We implement the adaptive dynamics framework to predict evolutionary outcomes in this model. We demonstrate that for simple trade-off functions between virulence, disease transmission and parasite growth rates, multiple evolutionary attractors are possible. Interestingly, unlike in the case of unstructured models, achieving an evolutionary stable strategy becomes possible even for a variation of a single ecological parameter (the parasite growth rate) and keeping the other parameters constant. We conclude that evolution in disease-structured populations is strongly mediated by alterations in the overall shape of the parasite load distribution.

**Electronic supplementary material:**

The online version of this article (10.1007/s00285-019-01351-6) contains supplementary material, which is available to authorized users.

## Introduction

Theoretical studies of the evolution of virulence and its control via research-driven management have a long history (Ebert and Weisser 1997; Lipsitch and Moxon 1997; Dieckmann 2002; Alizon et al. 2009; Morozov and Best 2012). Mathematical approaches to the problem have greatly developed, stemming from the original paradigmatic but the somewhat simplistic idea of maximisation of the basic reproduction number $R_0$ to modern advanced frameworks considering complex feedback loops between dynamical environment, the host and the pathogen (Dieckmann 2002; Lion and Metz 2018). Among other important factors, heterogeneity of host is currently considered to play a key role in evolution of virulence and transmission rate. The host heterogeneity can occur due to a variety of reasons including genetic variation, infection history, the overall physiological status (e.g. nutrition), age, sex, space and other factors (Dwyer et al. 1997; Keith and Mitchell-Olds 2013; Sorci et al. 2013) and it was shown to strongly affect the course of evolution, in particular resulting in evolutionary branching (Pugliese 2011; Lion and Metz 2018). The importance of considering the heterogeneity of host is closely related to the current trend in modelling in population dynamics which highlights the necessity of studying physiologically structured populations (Metz and Diekmann 1986; Calsina and Farkas 2014; Farkas 2018; Farkas and Hagen 2007; Magal et al. 2010; de Roos and Persson 2013).

On the other hand, the existing research into modelling the evolution of virulence has so far overlooked the fact that the host population is often structured in terms of parasite burden. Actually, this type of population structuring directly follows from the very nature of infection. Indeed, an infection in the organism can initiate with a small initial amount of parasites which will progressively grow inside the host in the course of time and will eventually result in the host’s death. Empirical data usually reveal the large variation of parasite load inside infected individuals both in domestic and wild animals (Hudson et al. 1992; Temple 1987; Craig et al. 2006; Klimpel et al. 2006). It was also reported that key characteristics such as virulence, transmission rate, reproduction and recovery rate are strongly dependent on the parasite load (Hudson et al. 1992; Craig et al. 2006) and this should be reflected in our theoretical approaches.

**Fig. 1:**
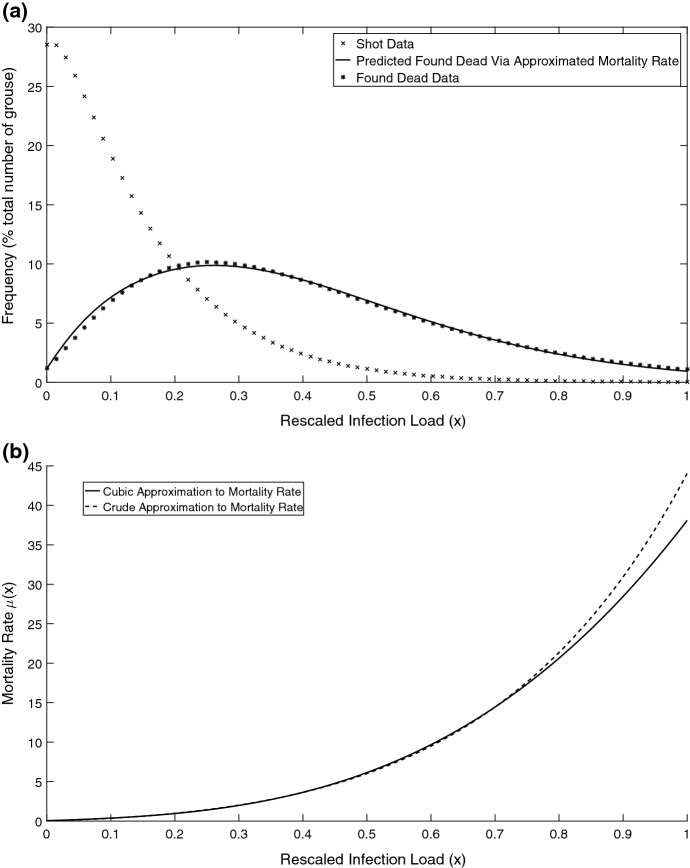
**a** Frequency distributions of infection load, as measured by worm concentration per bird, in red grouse population from data in Hudson et al. (1992) extracted from all study moors after smoothing initial data using the negative binomial distribution. The star symbols represent the frequency distribution of grouse that were already found dead whereas crosses show the distribution of grouse that were shot by hunters. The solid line represents an approximated distribution of infection load in birds found dead using the fitted mortality function (2.5) shown in panel **b** by a solid line. **b** Mortality rates approximated from the relationship between shot grouse and those that were found dead on the ground. The dashed line represents a crude approximation to the mortality displaying obtained by dividing the number of the shot individuals by the number found dead corresponding to the same load *x*. The solid line represents the mortality function based on a cubic approximation with parameters numerically fitted using the data such that the deviation between found dead data and predicted curve (‘*’ symbols and the solid line in panel **a**) would be minimal. The non-linear fit was done using MATLAB software

As an insightful case study, we briefly consider the infection of red grouse by a parasitic nematode in North England in 1980–84 using the data set from (Hudson et al. 1992). Figure 1a shows the frequency distribution of parasitic worms per bird. For simplicity, we re-scale the *x*-axis, so the maximum load $x=1$ corresponds to 17,000 worms per bird. Figure 1a also shows the parasite burden in birds found dead. One can clearly see a large variation in the parasite burden across the infected population. More interestingly, one can see that the distribution of dead birds is shifted (as compared to the distribution of infected) towards a higher infection load which can be explained by a strong increase in mortality with *x*. To estimate the effect of parasite load on mortality, we make a simple assumption that most of the birds found dead actually died recently before data collection. In this case, we plot a crude estimate of the dependence of the mortality of parasite number shown in Fig. 1b obtained as the number of dead individuals divided by the number of infected for the same *x* (a more accurate prediction should include the possibility of parasites growth inside dead bodies). We fitted a polynomial function (2.5) to the crude mortality data which is shown by a dashed line in Fig. 1b (for more details on extracting data and fitting the curves see the figure caption). From the figure, one can clearly see that the mortality rate dramatically increases with an increase in parasite load. It was also reported that birds with higher parasite loads had a higher probability to be consumed by their natural predator (Hudson et al. 1992).

We argue, however, that a major consequence for our mathematical models with host structuring in terms of parasite load is not the dependence of their life traits on the number of parasites in the body per se. Rather, the shape of the distribution of infected individuals as a function of parasite numbers can vary both on ecological and evolutionary scales which was confirmed by empirical data (Hudson et al. 1992). For example, for the same value of average parasite burden, different shapes of parasite load distributions would signify different overall mortality rates in the population. In this case, we cannot describe the overall distribution as the one in Fig. 1a by a single number (e.g. the average value of *x*). As such, the alteration of the overall shape of parasite burden should play as much role in the outcome of evolution as a variation of some scalar parameters as for example, the growth rate of the parasite. Finally, to support the necessity of considering models with distributed parasite loads, we should stress that only in some particular cases a continuous structured population model can be described via a finite-dimensional representation (Diekmann et al. 2017).

In this paper, we try to bridge the existing gap and explore the evolution of virulence in a host-parasite system with a distributed infection load. Mathematically, we extend the classical SI model: now the population of infected individuals is a continuous variable of infection load *x* described by a von Förster-type equation (Cushing 1998). In this case, the parasite load plays the role of age. We use the well-known adaptive dynamics framework (Geritz et al. 1997) to elaborate the evolutionary outcome of the system, where invasion fitness (determining the possibility of a rare mutant to invade the population of the resident strain) is given by an integro-differential equation. For the considered model with density-independent mortality, we prove that possible evolutionarily singular points can be either evolutionary attractors (Evolutionary Stable Strategies, ESSs) or repellors. Then we explore several insightful examples of evolutionary behaviour for particular biological trade-offs between key model parameters: mortality, parasite growth rate and transmission rate. We compare our results with a similar SI unstructured model.

We show that for simple trade-off scenarios with pairwise connections between mortality, parasite growth rate and transmission rate long-term evolution in the structured model may involve bistability: depending on initial parasite strain the parasite can evolve to a benign or a virulent strain. We also show that the evolution of a single life trait (growth rate of the parasite inside the host) can result in an ESS in the system, which was not possible for an unstructured model. Finally, we plot the emerging average trade-offs between the virulence and the overall transmission rate since such trade-off is frequently included in unstructured models often without a proper mechanistic explanation. We show that in systems with a distributed parasite load virulence-transmission dependence can demonstrate non-monotonicity: high values of virulence may reduce the overall transmission rate. This becomes possible due to a gradual shift of the distribution of the parasite load towards a lower infection burden.

## Model equations and general framework

The dynamics of the system are governed by the following equations of McKendrick–von Förster-type (Calsina and Farkas 2014, 2016)

2.1 $$\begin{aligned} \begin{aligned}&\frac{\mathrm {d}S}{\mathrm {d}t}(t) =r(S)S+\int _{x_{min}}^{x_{max}} \rho (x)i(x,t)\,dx-f(S)\int _{x_{min}}^{x_{max}}i(x,t) {\widetilde{{\varLambda }}}(x)\,dx,\\&i_{t}(x,t)+\left( \alpha (x)i(x,t)\right) _{x} =-\rho (x)i(x,t)-\mu (x)i(x,t), \end{aligned} \end{aligned}$$

with the boundary condition

2.2 $$\begin{aligned} \alpha (x_{min})i(x_{min},t)=f(S)\int _{x_{min}}^{x_{max}}i(x,t) {\widetilde{{\varLambda }}}(x)\,dx, \end{aligned}$$

and initial conditions

2.3 $$\begin{aligned} i(x,0)=i_{0}(x),\quad S(0)=S_{0}. \end{aligned}$$

This model is actually an extension of a classic susceptible-infected (SI) host-parasite model where the variables describe the densities of healthy individuals (*S*) and infected individuals (*i*), i.e. those carrying parasites. Infected individuals *i* are structured with respect to the parasitic load *x*. We assume that individuals are firstly infected by some minimal parasite load $x_{min}$. The maximal infection load is denoted by $x_{max}$. Note that we can always shift and re-scale *x* such that the range $x\in [x_{min},x_{max}]$ becomes $x\in [0,1]$. Thus, from hereafter we suggest that $x_{min}=0$ and $x_{max}=1$.

The reproduction of susceptible members of the population is described by the per capita growth rate *r*(*S*): here we assume that infected individuals do not reproduce. In illustrative examples (Sect. 3.3) we consider *r*(*S*)*S* to be as a logistic growth, i.e. $r(S)=r_0(1-S/K)$ (Tsoularis and Wallace 2002), where $r_0$ stands for the combination of the density-dependent highest per capita growth and the density-independent per capita mortality $\mu _0$, with *K* being the carrying capacity. The infection process is described by the boundary condition (2.2). The probability that susceptible individuals become infected by contacting infected ones is proportional to *f*(*S*), which is assumed to be linear in the examples we consider in Sect. 3.3. However, *f* can generally be a non-linear function as well accounting for possible superlinear or sublinear spread of infection (Hochberg 1991; Adler and Mosquera Losada 2002). To obtain the contribution of the whole subpopulation of infected into disease transmission, one needs to integrate the elementary transmission term $f(S)i(x,t){\widetilde{{\varLambda }}}(x)$ over $x\in [0,1]$. Here is ${\widetilde{{\varLambda }}}(x)$ the transmission rate corresponding to infection load *x*.

The terms $\rho (x)i(x,t)$ and $\mu (x)i(x,t)$ in (2.1) describe, respectively, the recovery rate of infected individuals and their death rate. Finally, the function $\alpha (x)$ stands for the increase in parasite numbers within an individual due to reproduction and consumption of the host.

In this paper, we are interested in exploring the evolution of parasite life traits: which are the mortality $\mu$, the infection growth $\alpha$, the recovery rate $\rho$ and the transmission rate ${\widetilde{{\varLambda }}}$, all of which are functions of *x* in general. Clearly, various scenarios are possible, for example where some (or all) those traits might be fully independent of each other. However, here for the sake of simplicity, we assume that each of them depends on a certain parameter $\epsilon$ which can be characterised by a parasite strength or (fitness). Biologically, this can be, for example, the reproduction rate of parasites within the host, parasite maturation time, parasite survival rate (e.g. resistance to the host immune system) or similar traits which we can measure in our experiments. For simplicity, we assume that $\mu$, $\alpha$, $\rho$ and ${\widetilde{{\varLambda }}}$ are functions of only one parameter $\epsilon$. This does not exclude the particular case where some trait does not evolve and remains constant. In the course of evolution, $\epsilon$ changes with time, thus resulting in the change of other traits. In this study, we assume (using biological common sense) that the growth of parasite load is an increasing function of $\epsilon$; it is also logical to suggest that $\alpha (0,\epsilon )\ge 0$ and $\alpha (x,0)=0$. In other words, the initial growth rate at the minimal parasite load is positive and if the parasite strength vanishes, its growth is not possible anymore. In numerical examples (Sect. 3.3) we consider that

2.4 $$\begin{aligned} \alpha (x,\epsilon )= g(\epsilon )((x-B_1)^2(x+A_1)\exp (-D_1(x+A_1))+C_1), \end{aligned}$$

where $A_1$, $B_1$, $C_1$ and $D_1$ are positive parameters. We choose $A_1$ close to 0 and $B_1$ close to 1. For parameters considered here this function has a unique maximum on (0, 1). Apart from the above parametrisation, we consider other functional dependencies (see Sect. 4). Here $g(\epsilon )$ is a function describing the link between the parasite strength and the growth rate. In the supplementary material, we provide an example plot of this function.

The mortality rate $\mu (x,\epsilon )$ is parameterised here by the following polynomial function which provides a good approximation to some data (Fig. 1b).

2.5 $$\begin{aligned} \mu (x,\epsilon )=\mu _{0}+h(\epsilon )(Ax+Bx^{2}+Cx^{3}), \end{aligned}$$

where $\mu _0$ is the background (i.e. disease-independent) mortality. An increase in the strength of infection $\epsilon$ increases the mortality term which depends on the parasite load and it is an increasing function with respect to *x*, with *A*, *B*, *C* being positive constants. Here $h(\epsilon )$ is a function describing the link between the parasite strength and the extra mortality.

The model also allows for possible recovery of infected individuals returning back into their healthy susceptible state, described by the function $\rho (x,\epsilon )$. This function should be decreasing with respect to both the infection load and the evolutionary parameter. As a particular parametrisation, we consider the following form

2.6 $$\begin{aligned} \rho (x,\epsilon )=a_{\rho }(\epsilon )\left( 1-x^{2}\right) \end{aligned}$$

where $a_{\rho }$ is some scaling constant. In our theoretical results we assume a general form for $\rho (x,\epsilon )$, however, in all of the numerical examples in Sect. 3.3 we assume that $a_{\rho }(\epsilon )=const$, i.e. the recovery rate does not depend on the parasite strength.

Here we do not admit the co-existence of multiple strains of the parasite within a single host (the case of co-infection should be considered elsewhere).

Finally, we assume that the function determining the transmission rate ${\widetilde{{\varLambda }}}(x,\epsilon )$ increases with respect to both *x* and $\epsilon$; moreover, we require that ${\widetilde{{\varLambda }}}(0,\epsilon )=0$ and ${\widetilde{{\varLambda }}}(x,0)=0$ holds. For simplicity, we consider a parametrisation where ${\widetilde{{\varLambda }}}(x,\epsilon )$ is given by the product of the function $V(\epsilon )$ (depending only on the strength of infection) and the function ${\varLambda }(x)$ which describes the dependence on the parasite load. One can suggest various parameterisations of $V(\epsilon )$ and their choice might have a strong effect on the evolutionary outcome (de Mazancourt and Dieckmann 2004; Hoyle et al. 2008). Following the studies of the evolution of virulence in unstructured populations (Dieckmann 2002), we consider the following possibilities: no trade-off, a linear dependence and a Monod (hyperbolic)-type function. Note that the non-linear trade-off is a concave function as it is frequently assumed in the literature. We also explore the simplest case where *V* does not depend on $\epsilon$. These parametrisations are listed in Table 1. Note that in most numerical examples we consider $g(\epsilon )=\epsilon$ and $h(\epsilon )=\epsilon$ in (2.5) and (2.4) unless it is stated otherwise.

**Table 1:** Different parametrisations of the transmission rate *V* depending on the strength of the parasite characterised by $\epsilon$

Parametrisation type	$V(\epsilon )$
No trade-off	$V(\epsilon )= \hbox {constant}$
Linear	$V(\epsilon )=c\epsilon$
Simple Monod (hyperbolic)	$V(\epsilon )=c\frac{\epsilon }{\epsilon +k}$

The coefficients *c* and *k* are positive

We consider that the term ${\varLambda }(x)$ describing the parasite load dependence of the transmission rate is given by

2.7 $$\begin{aligned} {\varLambda }(x)={\varLambda }_{0}-a_{{\varLambda }}(k-x)^m, \end{aligned}$$

where ${\varLambda }_{0}$ is the maximal possibility of transmission chosen such that ${\varLambda }(0)=0$ with $a_{{\varLambda }}$, *m* and *k* being some positive constants.

In our simulated examples in Sect. 3.3 we explore pairwise connections of trade-offs between the functions *V*, $\alpha$ and $\mu$, keeping a third trait constant. In other words, we explore the following three cases: (i) $V(\epsilon )$, $\mu (x,\epsilon )$ with $\alpha (x)$; (ii) $V(\epsilon )$, $\alpha (x,\epsilon )$ with $\mu (x)$ and (iii) $\alpha (x,\epsilon )$, $\mu (x,\epsilon )$ with a constant *V*.

The evolution of life traits in the model was investigated using the adaptive dynamics framework (Geritz et al. 1997; Brännström et al. 2013). The basic concept of adaptive dynamics is a separation of time scales, and considering the possibility of the invasion of a rare mutant into the environment formed by the resident at ecological equilibrium. The long-term evolutionary outcome is characterised by the so-called invasion fitness: a successful invasion would signify a positive fitness of the invader (Eshel 1983; Taylor 1989; Christiansen 1991; Abrams et al. 1993). As a result of a large number of invasions and substitutions of resident populations by mutants, the pathogen strain will eventually evolve until it reaches an evolutionary singular point, at which the selection gradient vanishes. The further evolutionary behaviour will depend on the stability of evolutionary singularities, i.e. ‘stopping points’ (Taylor 1989; Abrams et al. 1993). In adaptive dynamics, an evolutionary attractor signifies that a singular point should be both an evolutionary stable strategy (ESS)—the nearby mutants are not able to invade—and convergent stable strategy ensuring that an ESS can be attained (Eshel 1983). The evolutionary stability (or instability) can be determined from either a Pairwise Invasibility Plot (Kisdi and Meszena 1993) (PIP), and/or by computing the second derivatives of the invasion fitness at a singular point (Eshel 1983; Geritz et al. 1998). Finally, in the case of convergence of the evolutionary trajectory to a singular point which is not an ESS, a dimorphism can occur and results in branching (Geritz et al. 1998). Note that analytical verification of singular points is only possible in the case where the ecological attractor is a stable equilibrium.

From the adaptive dynamics point of view the main challenge of using the structured model (2.1)–(2.3) is that the invasion fitness can only be characterised implicitly as a leading eigenvalue of an (unbounded) linear operator; and to obtain detailed information one typically has to resort to numerical techniques (see the subsequent section for details). To verify stability conditions for the stationary state we use the corresponding characteristic equation and apply in-depth numerical techniques (Ames 2014; Davis and Rabinowitz 2007). Moreover, we also conduct direct numerical simulations of the model equation to follow the invasion of mutants and replacement of the resident. The computation is based on a finite difference scheme using upwind discretisation (Abia et al. 2005).

## Results

### Stationary states and their stability

It is clear that model (2.1) with boundary condition (2.2) admits the disease-free trivial stationary state $(0,\widetilde{S_*})$ where all members of the population are healthy. Here $\widetilde{S_*}$ is the solution of the equation $r(\widetilde{S_*})=0$, thus in the case of the logistic growth function we have that $S_*$ is equal to the carrying capacity. The stability of this equilibrium determines whether or not a parasite strain characterised by $\epsilon$ can invade the initially healthy population *S* at the carrying capacity. The condition of parasite spread is given by the following

#### Proposition 1

A parasite strain $\epsilon$ can successfully spread in a fully susceptible population at $\widetilde{S_*}$ provided that the basic reproduction number

3.1 $$\begin{aligned} R_0(\epsilon )=f(\widetilde{S_*})V(\epsilon )\intop _{0}^{1} \exp \left( -\intop _{0}^{x}\frac{\rho (y,\epsilon )+\mu (y,\epsilon )}{\alpha (y,\epsilon )}\,dy\right) \frac{{\varLambda }(x)}{\alpha (x,\epsilon )} \,dx \end{aligned}$$

is greater than one. Otherwise, the parasite will go extinct. Therefore, the range of viable $\epsilon$ values to be considered further to determine possible evolutionary outcomes is determined by the condition $R_0(\epsilon )>1$.

This proposition can be established following standard techniques, similar to that those applied to age-structured population models (Li and Brauer 2008; Martcheva 2015). Note that the exponential kernel inside the integral is due to the fact that the underlying model uses structured partial differential equations. The full details of this can be found in the supplementary material.

The model has a strictly positive steady state $\left( i_{*}(x),S_{*}\right)$, subject to the parameters satisfying certain constraints. To find an analytic expression for $i_{*}(x)$, we solve the first equation of (2.1) for a time-independent solution to obtain

3.2 $$\begin{aligned} \begin{aligned} i_{*}(x)=\,&i_{*}(0)\frac{\alpha (0,\epsilon )}{\alpha (x,\epsilon )}\exp \left\{ -\int _{0}^{x}\frac{\rho (y,\epsilon )+\mu (y,\epsilon )}{\alpha (y, \epsilon )}\,dy\right\} \\ =\,&i_{*}(0)\exp \left\{ -\int _{0}^{x}\frac{\rho (y,\epsilon )+ \mu (y,\epsilon )+\alpha '_{y}(y,\epsilon )}{\alpha (y,\epsilon )}\,dy\right\} . \end{aligned} \end{aligned}$$

Note that we have expanded our model (2.1)–(2.3) to account for parameters $\rho , \mu , \alpha , {\widetilde{{\varLambda }}}$ depending on $\epsilon$, too, as detailed in the previous section. Substituting expression (3.2) for $i_*(x)$ into the boundary condition (2.2), we obtain the following implicit formula for $S_*$

3.3 $$\begin{aligned} 1=f(S_{*})V(\epsilon )\int _{0}^{1}\exp \left\{ -\int _{0}^{x}\frac{\rho (y,\epsilon )+\mu (y,\epsilon )}{\alpha (y,\epsilon )}\,dy\right\} \frac{{\varLambda }(x)}{\alpha (x,\epsilon )}\,dx. \end{aligned}$$

Assuming that *f*(*S*) is monotonically increasing, it is possible to find a unique $S_{*}$ value for each $\epsilon$ such that (3.3) holds. Then, the value of $S_*$ can be substituted into the second equation of (2.1), yielding the expression for $i_{*}(0)$

3.4 $$\begin{aligned} i_{*}(0)=\frac{r(S_{*})S_{*}}{\alpha (0,\epsilon )\left( 1-{\displaystyle \int _{0}^{1}\frac{\rho (x,\epsilon )}{\alpha (x,\epsilon )}\exp \left\{ -{\displaystyle \int _{0}^{x}\frac{\rho (y,\epsilon ) +\mu (y,\epsilon )}{\alpha (y,\epsilon )}\,dy}\right\} dx}\right) },\nonumber \\ \end{aligned}$$

which then uniquely determines $i_*(x)$ via (3.2).

We summarize our findings in the proposition below, by establishing conditions which guarantee the existence of a positive steady state.

#### Proposition 2

Assume that

3.5 $$\begin{aligned} f(0)=0,\quad f'(S)\ge 0,\quad f(S)\rightarrow \infty \,\,\text {as}\,\,S\rightarrow \infty , \quad r (S_*)>0, \end{aligned}$$

holds, where $S_*$ is determined by (3.3). Then, for every $\epsilon \in {\mathbb {R}}_+$ such that

3.6 $$\begin{aligned} 1>\int _0^1\frac{\rho (x)}{\alpha (x,\epsilon )} \exp \left\{ -\int _0^x \frac{\rho (y,\epsilon )+\mu (y,\epsilon )}{\alpha (y,\epsilon )}\, dy\right\} dx, \end{aligned}$$

holds true, model (2.1) admits a positive steady state $(i_*(x),S_*)$.

#### Stability of the positive stationary state

Linearising (2.1) at a steady state $(i_*(x),S_*)$ we obtain:

3.7 $$\begin{aligned} j_t(x,t) + \left( \alpha (x,\epsilon )j(x,t)\right) _x&=\,&-\rho (x,\epsilon )j(x,t)-\mu (x,\epsilon )j(x,t), \nonumber \\ \alpha (0,\epsilon )j(0,t)&=\,&f(S_*)\int _0^1 j(x,t){\varLambda }(x) V(\epsilon )\,\mathrm {d}x \nonumber \\&+\, U(t)f'(S_*)\int _0^1 i_*(x){\varLambda }(x)V(\epsilon )\,\mathrm {d}x\nonumber \\ U'(t)&=\,&r(S_*)U(t) + r'(S_*)S_*U(t)+ \int _0^ 1\rho (x,\epsilon )j(x,t)\,\mathrm {d}x \nonumber \\&- \,f(S_*)\int _0^1 j(x,t){\varLambda }(x) V(\epsilon )\,\mathrm {d}x \nonumber \\&-\,U(t)f'(S_*)\int _0^1 i_*(x){\varLambda }(x)V(\epsilon )\,\mathrm {d}x.\nonumber \\ \end{aligned}$$

The linearised model (3.7) is formally a special case of the linearisation of a general *Daphnia* (structured consumer-resource) model we obtained in Farkas and Hagen (2007), hence we may invoke Theorem 2.1 from (Farkas and Hagen 2007), which guarantees the existence of a strongly continuous semigroup on the state space ${\mathcal {X}}=L^1(0,1)\times {\mathbb {R}}$ with norm $||\cdot ||_{\mathcal {X}}=||\cdot ||_{L^1}+|\cdot |$, governing the linearised model (3.7). Furthermore, Lemma 2.2 in Farkas and Hagen (2007) implies that the asymptotic behaviour of (3.7) is governed by eigenvalues of the semigroup generator. Also, note that our model (2.1) is semi-linear and therefore the local stability of steady states is indeed determined by the asymptotic behaviour of the linear semigroup governing the linearised equations, for example see the stability principles established in Henry (1981, Ch. 5).

The linearisation (3.7) at the strictly positive steady state $(i_*(x),S_*)$ leads to an eigenvalue problem. In particular $\lambda \in {\mathbb {C}}$ is an eigenvalue if and only if the following two-dimensional homogeneous system—for the variables $({\bar{j}}(0),{\bar{U}})$—admits a non-trivial solution.

3.8 $$\begin{aligned} \begin{aligned} 0=\,&{\bar{j}}(0)\left( f(S_*)V(\epsilon )\int _0^1{\varLambda }(x){\varPi }(x,\lambda )\,\mathrm {d}x-\alpha (0,\epsilon )\right) \\&+{\bar{U}}f'(S_*)V(\epsilon )\int _0^1i_*(x) {\varLambda }(x)\,\mathrm {d}x \\ 0=\,&{\bar{j}}(0)\left( \int _0^1\rho (x,\epsilon ){\varPi }(x,\lambda )\,\mathrm {d}x-f(S_*)V(\epsilon )\int _0^1{\varLambda }(x){\varPi }(x,\lambda )\,\mathrm {d}x \right) \\&+ {\bar{U}}\left( r(S_*)+r'(S_*)S_*- \lambda -f'(S_*)V(\epsilon ) \int _0^1i_*(x){\varLambda }(x)\,\mathrm {d}x \right) . \end{aligned} \end{aligned}$$

Above we introduced the notation

3.9 $$\begin{aligned} {\varPi }(x,\lambda )= & {} \exp \left\{ -\int _0^x\frac{\lambda +\rho (y,\epsilon ) +\mu (y,\epsilon )+\alpha '_{y}(y,\epsilon )}{\alpha (y,\epsilon )}\,\mathrm {d}y\right\} , \nonumber \\&\quad x\in [0,1],\quad \lambda \in {\mathbb {C}}. \end{aligned}$$

By setting the determinant of the homogeneous system (3.8) to equal zero, a characteristic equation for $\lambda$ can be deduced. After some simplification, it can be written in the most economical form as

3.10 $$\begin{aligned}&(r(S_*)+r'(S_*)S_*-\lambda )\left( f(S_*)V(\epsilon ) \int _0^1{\varLambda }(x){\varPi }(x,\lambda )\,\mathrm {d}x-\alpha (0,\epsilon )\right) \nonumber \\&\quad = \frac{f'(S_*)}{f(S_*)}i_*(0)\alpha (0,\epsilon ) \left( \int _0^1\rho (x,\epsilon ){\varPi }(x,\lambda )\,\mathrm {d}x-\alpha (0,\epsilon )\right) . \end{aligned}$$

We note that the semigroup governing the linearised model (3.7) can be shown positive [see Theorem 2.3 in Farkas and Hagen (2007)] if the following conditions hold true:

3.11 $$\begin{aligned} f'(S_*)\ge 0,\quad \rho (x,\epsilon )\ge f(S_*){\varLambda }(x)V(\epsilon ),\,\, \forall \,x\in [0,1]. \end{aligned}$$

However, note that condition (3.11) cannot hold true if a positive steady state exists (this is due to the negative feedback mechanism in model (2.1)). Indeed, the stability criterion we formulated in Theorem 3.1 in Farkas and Hagen (2007) for the more general model cannot hold true; and as we noted in Remark 3.5 in Farkas and Hagen (2007), stability is not necessarily governed by a leading real eigenvalue. Hence to establish the local asymptotic stability of the positive steady state $(i_*(x),S_*)$ we need to guarantee that the characteristic equation (3.10) does not admit a solution $\lambda \in {\mathbb {C}}$ with Re$(\lambda )\ge 0$, in general. We are going to prove this first for the special case when $\rho (x,\epsilon )\equiv \rho$ and ${\varLambda }(x)\equiv {\varLambda }$.

##### Proposition 3

Assume that $f'(S_*)>0$ and $r(S_*)+r'(S_*)S_*<0$ hold, and that $\rho (x,\epsilon )\equiv \rho$ and ${\varLambda }(x)\equiv {\varLambda }$. Then, if model (2.1) admits a positive steady state $(i_*(x),S_*)$, it is locally asymptotically stable.

##### Proof

For brevity let us introduce the following notation

$$\begin{aligned} {\varOmega }:=f'(S_*)V(\epsilon ){\varLambda }\int _0^1i_*(x)\, \mathrm {d}x, \quad {\varTheta }:=r(S_*)+r'(S_*)S_*,\quad {\varGamma }(x):=\int _0^x\frac{\mathrm {d}y}{\alpha (y,\epsilon )}. \end{aligned}$$

Using the notation above we rewrite the characteristic equation (3.10) as

3.12 $$\begin{aligned}&{\varOmega }\,\rho \int _0^1{\varPi }(x,\lambda )\,\mathrm {d}x-{\varOmega }\,\alpha (0,\epsilon ) \nonumber \\&\quad = ({\varTheta }-\lambda ) \left( f(S_*)V(\epsilon ){\varLambda }\int _0^1{\varPi }(x,\lambda )\,\mathrm {d}x-\alpha (0,\epsilon )\right) . \end{aligned}$$

Equation (3.12) can be rewritten as

3.13 $$\begin{aligned} \frac{f(S_*)V(\epsilon ){\varLambda }}{\alpha (0,\epsilon )}\int _0^1 {\varPi }(x,\lambda )\,\mathrm {d}x=\frac{\lambda -{\varTheta }+{\varOmega }}{\lambda -{\varTheta }+{\varOmega }\frac{\rho }{f(S_*)V(\epsilon ){\varLambda }}}. \end{aligned}$$

Taking the real part on both sides of equation (3.13) we obtain the equality

3.14 $$\begin{aligned}&\frac{f(S_*)V(\epsilon ){\varLambda }}{\alpha (0,\epsilon )} \int _0^1{\varPi }(x,0)\exp \left\{ -\text {Re}(\lambda ){\varGamma }(x) \right\} \cos \left( \text {Re}(\lambda ){\varGamma }(x)\right) \,\mathrm {d}x \nonumber \\&\quad = \frac{\left( \text {Re}(\lambda )- {\varTheta }+{\varOmega }\right) \left( \text {Re}(\lambda )-{\varTheta }+{\varOmega }\frac{\rho }{f(S_*) V(\epsilon ){\varLambda }}\right) +\text {Im}(\lambda )^2}{\left( \text {Re}(\lambda )-{\varTheta }+{\varOmega }\frac{\rho }{f(S_*)V(\epsilon ){\varLambda }}\right) ^2+ \text {Im}(\lambda )^2}. \end{aligned}$$

Note that since ${\varGamma }(x)>0,\,\forall x\in (0,1)$, the left hand side of equation (3.14) is less than or equal to 1 for any $\lambda \in {\mathbb {C}}$ with Re$(\lambda )\ge 0$. On the other hand, since ${\varOmega }>0,\, {\varTheta }<0$ and $\rho <f(S_*)V(\epsilon ){\varLambda }$ holds (if there exists a positive steady state), the right hand side of (3.14) is greater than 1 for any $\lambda \in {\mathbb {C}}$ with Re$(\lambda )\ge 0$. Hence we conclude that the characteristic equation (3.10) does not admit a solution $\lambda \in {\mathbb {C}}$ with Re$(\lambda )\ge 0$, and therefore the positive steady state $(i_*(x),S_*)$ is locally asymptotically stable. $\square$

Next we formulate a sufficient condition for stability, which applies to more general model parameters, i.e. when $\rho$ and ${\varLambda }$ are not necessarily constant; but at the same time it is more restrictive.

##### Proposition 4

Suppose that model (2.1)–(2.3) admits a positive steady state $(i_*(x),S_*)$, and that

3.15 $$\begin{aligned} r(S_*)+r'(S_*)S_*<0,\,\,\, f'(S_*)\ge 0, \,\,\, 2f(S_*){\varLambda }(x)V(\epsilon )<\mu (x,\epsilon ),\,\,\forall \,x\in [0,1],\nonumber \\ \end{aligned}$$

hold true. Then the steady state $(i_*(x),S_*)$ is locally asymptotically stable.

This result is established using a quasi-dissipativity approach (which does not rely on positivity or on the existence of a characteristic equation), and details of the proof can be found in Appendix A.

We would like to note that for a large number of our numerical simulations (see Sect. 3.3), the stability condition $2f(S_*){\varLambda }(x)V(\epsilon )<\mu (x,\epsilon )$ does not hold true, thus we we could not invoke the stability criterion directly. Nevertheless, for these examples, we numerically solved the characteristic equation (3.10) to check the sign of the the real part of the dominant eigenvalue. Note however that within all of the range of the parameters we considered, the positive stationary state (where it existed) was indeed shown stable.

### Evolutionarily singular points and their properties

We first derive the expression for invasion fitness which is crucial in the theory of adaptive dynamics. Suppose that there is some resident population at its stationary state where the parasite strain is characterised by $\epsilon =\epsilon _{r}$ and the size of the susceptible subpopulation $S_{*}$ is determined by Eq. (3.3). Now suppose that a very small quantity of a mutant population *m*(*x*, *t*) characterised by $\epsilon =\epsilon _{m}$ is introduced into this resident environment. By the main hypothesis of adaptive dynamics (Geritz et al. 1998), the dynamics of this mutant population can be described by the following linear model

3.16 $$\begin{aligned} \begin{aligned} m_{t}(x,t)+\left( \alpha (x,\epsilon _{m})m(x,t)\right) _{x}&=-\rho (x,\epsilon _m)m(x,t)-\mu (x,\epsilon _{m})m(x,t),\\ \alpha (0,\epsilon _{m})m(0,t)&=f(S_{*})\int _{0}^{1}m(x,t){\varLambda }(x)V(\epsilon _{m})\,dx, \end{aligned} \end{aligned}$$

together with the initial condition

3.17 $$\begin{aligned} m(x,0)=m_{0}(x)\ll i_{*}(x), \end{aligned}$$

where $S_{*}$ and $i_{*}$ are the stationary states determined by (3.3) and (3.2), respectively, and assuming the parasite is evolving.

As model (3.16) describing the dynamics of the mutant population is a linear partial differential equation governed by an eventually compact semigroup, for any fixed $\epsilon _{m}$ and $\epsilon _{r}$ (and hence for any fixed $S_{*}$), the invasion fitness can be found as the leading eigenvalue of the generator of the semigroup, corresponding to model (3.16) governing the invading parasite strain *m*. To determine this leading eigenvalue we look for solutions of (3.16) in the following form

$$\begin{aligned} m(x,t)=\exp \left( \lambda (\epsilon _{r},\epsilon _{m})\,t\right) J(x), \end{aligned}$$

where $0\not \equiv J\in W^{1,1}(0,1)$ (the Sobolev space of absolutely continuous functions). After some straightforward calculations we obtain the following (implicit) expression for the invasion fitness $\lambda (\epsilon _{r},\epsilon _{m})$

3.18 $$\begin{aligned} \frac{\displaystyle \int _{0}^{1}\frac{{\widetilde{{\varLambda }}}(x,\epsilon _{m})}{\alpha (x,\epsilon _{m})}\exp \left( -\displaystyle \int _{0}^{x}\frac{\lambda (\epsilon _{r},\epsilon _{m})+\rho (y,\epsilon _{m})+\mu (y, \epsilon _{m})}{\alpha (y,\epsilon _{m})}\,dy\right) \,dx}{\displaystyle \int _{0}^{1}\frac{{\widetilde{{\varLambda }}}(x,\epsilon _{r})}{\alpha (x,\epsilon _{r})}\exp \left( -\displaystyle \int _{0}^{x} \frac{\rho (y,\epsilon _{r})+\mu (y,\epsilon _{r})}{\alpha (y,\epsilon _{r})}\,dy\right) \,dx}=1.\qquad \end{aligned}$$

It is possible to use numerical techniques to solve equation (3.18) for $\lambda (\epsilon _{r},\epsilon _{m})$, and therefore it can be used to determine the sign of the invasion fitness $\lambda$, and in turn to determine if the invasion of the mutant will be successful or not. We can analytically find derivatives of $\lambda (\epsilon _{r},\epsilon _{m})$ (as the left hand side of (3.18) is a smooth function) to determine evolutionarily singular points (satisfying $\lambda '=0$) and study their stability. When searching for singular points, we verify that $R_0(\epsilon )>1$ holds. Once all singular points have been located, their stability can be analysed. The condition for ESS-stability is that $\frac{\partial ^{2}\lambda (\epsilon _{r},\epsilon _{m})}{\partial \epsilon _{m}^{2}}<0$ holds, whereas the condition for convergence stability is that $\frac{\partial ^{2}\lambda (\epsilon _{r},\epsilon _{m})}{\partial \epsilon _{r}^{2}}-\frac{\partial ^{2}\lambda (\epsilon _{r},\epsilon _{m})}{\partial \epsilon _{m}^{2}}>0$ holds. Clearly, to verify either of these stability conditions the derivative of the leading eigenvalue $\lambda (\epsilon _r,\epsilon _m)$ has to be computed at the considered singular point.

The results of our investigation of possible types of singular points are summarised as follows.

#### Theorem 1

Suppose that there exists a singular point corresponding to a stable stationary state of model (2.1)–(2.2). Let the model parameters $\mu$, $\alpha$, $\rho$ and ${\widetilde{{\varLambda }}}$ be smooth functions of the evolutionary parameter $\epsilon$. Then this singular point has convergence stability if and only if it has ESS-stability, hence the only possible types of evolutionary outcomes are an evolutionary attractor or an evolutionary repellor with branching being impossible.

Note that the above theorem holds for the generic case where $\mu$, $\alpha$, $\rho$ and ${\widetilde{{\varLambda }}}$ can all be functions of $\epsilon$, still assuming that a singular point exists in this case. Moreover, the theorem holds for any shape of ${\widetilde{{\varLambda }}}$, i.e. not necessarily only for separable functions, i.e. ${\widetilde{{\varLambda }}}=V(\epsilon ){\varLambda }(x)$, which case we will investigate further in our numerical examples. The proof can be derived from direct computations of derivatives which, however, would result in cumbersome expressions. Here, for the sake of simplicity, we address the case where only $\mu$ and *V* are both dependent on the evolutionary parameter $\epsilon$. The proof for the general statement can be established following similar lines.

#### Proof

We compute the first and the second derivatives by differentiating the invasion fitness given by (3.18) with respect to $\epsilon _{r}$ and $\epsilon _{m}$. The derivation of the first and the second derivatives can be found in Appendix B. Comparing the two derivatives $\frac{\partial ^{2}\lambda (\epsilon _{r},\epsilon _{m})}{\partial \epsilon _{m}^{2}}$ and $\frac{\partial ^{2}\lambda (\epsilon _{r},\epsilon _{m})}{\partial \epsilon _{r}^{2}}$ it is clear that following equality holds.

3.19 $$\begin{aligned} \frac{\partial ^{2}\lambda (\epsilon _{r},\epsilon _{m})}{\partial \epsilon _{m}^{2}}=-\frac{\partial ^{2}\lambda (\epsilon _{r}, \epsilon _{m})}{\partial \epsilon _{r}^{2}} \end{aligned}$$

From this it can be concluded that if the singular point is ESS-stable (Geritz et al. 1997; Brännström et al. 2013), that is, $\frac{\partial ^{2}\lambda (\epsilon _{r}, \epsilon _{m})}{\partial \epsilon _{m}^{2}}<0$, which holds if and only if $\frac{\partial ^{2}\lambda (\epsilon _{r}, \epsilon _{m})}{\partial \epsilon _{r}^{2}}-\frac{\partial ^{2} \lambda (\epsilon _{r},\epsilon _{m})}{\partial \epsilon _{m}^{2}}>0$, then the singular point is also convergence stable, therefore this point is an evolutionary attractor. On the other hand if the singular point is not ESS-stable, that is, $\frac{\partial ^{2}\lambda (\epsilon _{r},\epsilon _{m})}{\partial \epsilon _{m}^{2}}>0$, which is true if and only if $\frac{\partial ^{2}\lambda (\epsilon _{r},\epsilon _{m})}{\partial \epsilon _{r}^{2}}-\frac{\partial ^{2}\lambda (\epsilon _{r},\epsilon _{m})}{\partial \epsilon _{m}^{2}}<0$, then the singular point is not convergence stable, therefore this point is an evolutionary repellor. Hence a singular point is either both ESS-stable and convergence stable or it is neither, therefore branching can never occur. $\square$

#### Remark 1

Using a similar approach one can prove the absence of branching points in the classical SI model without structuring with respect to the parasite load. The main requirement is that the mortality of infected individuals is density independent, that is, when $\mu =\mu _0$.

### Evolutionary outcomes in the system

Using the expressions for invasion fitness and its derivatives at evolutionarily singular points, one can explore possible evolutionary outcomes in the system. The goal of this subsection is to provide several examples of evolutionary behaviour with insightful biological interpretations rather than to give an exhaustive list of all possible regimes. Below we consider pairwise evolutionary connections between two out of three functions $\mu (x,\epsilon )$, $V(\epsilon )$ and $\alpha (x,\epsilon )$, the recovery rate $\rho (x,\epsilon )$ being always fixed.

#### Trade-off between mortality $\mu$ and transmission rate *V*

We start here with the trade-off dependence which was central in previous studies of virulence evolution in the population with unstructured parasite load (Alizon et al. 2009; Lipsitch and Moxon 1997; Ebert and Bull 2003). This involves the link between infection-induced mortality (virulence) and the transmission rate. The main difference between the previous studies is that here the transmission rate is not an explicit function of virulence, but is linked via the parasite strength $\epsilon$. We analyse various shapes of trade-off functions $V(\epsilon )$ from Table 1. For simplicity we assume that $h(\epsilon )=\epsilon$.

**Fig. 2:**
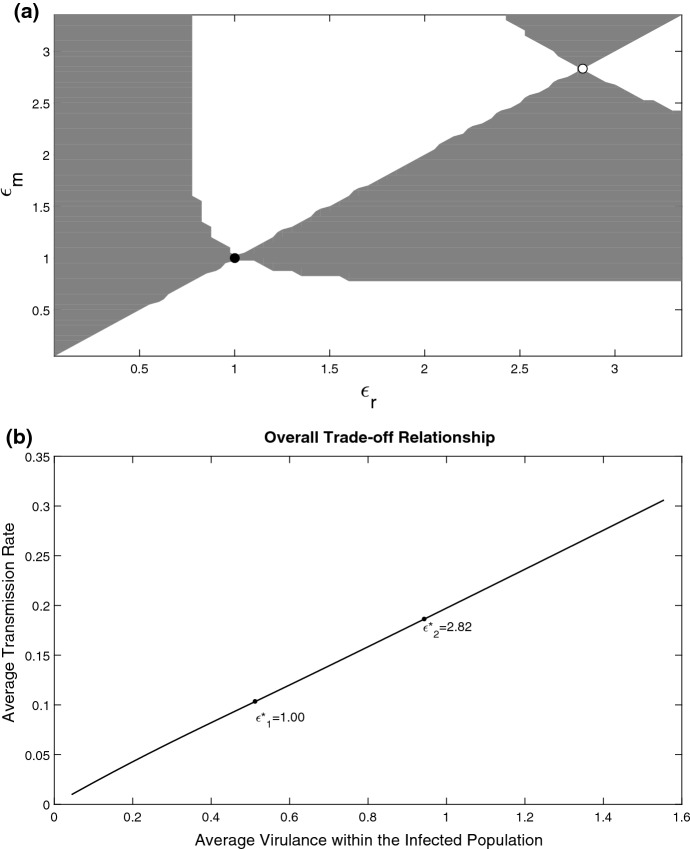
**a** Pairwise Invasibility Plot (PIP) describing long-term evolution in the system with $$V(\epsilon )=c\epsilon $$, $$h(\epsilon )=\epsilon $$ in $$\mu (x,\epsilon )$$. Here $$g(\epsilon )\equiv 1$$. Invasion fitness is plotted in the plane $$(\epsilon _{r},\epsilon _{m})$$ using (3.18). The grey regions represent a positive invasion fitness and the white regions represent a negative invasion fitness. The two evolutionary singular strategies are the evolutionary attractor shown by a backfilled circle and an evolutionary repellor shown by the open circle. **b** Overall trade-off relationship between the average virulence and the average transmission rate across the parasite burden. The trade-off is computed for varying values of the evolutionary parameter $$\epsilon $$ within the viable infection range. The singular points are shown by the two black points and correspond to those found in panel **a**. Other parameters are $$B_1=0.95$$, $$A_1=0.05$$, $$C_1=0.1$$, $$D_1=5$$, $$a_{\rho }=0.05$$, $$A=0.01$$, $$B=1$$, $$C=0.4$$, $$D=0.1$$, $$\mu _0=0.1$$, $$m=0.4$$$${\varLambda }_0=1$$, the function *f*(*S*) is given by $$f(S)=0.5S$$

*No trade-off* Initially consider the simplest case of no trade-off, i.e. where *V* does not depend on $\epsilon$. One can analytically prove by differentiating invasion fitness that in this case no (nontrivial) singular point is feasible with all mutations evolving towards the minimum plausible value of $\epsilon$ due to the mortality’s dependence on $\epsilon$. Note that the same conclusion is made in an SI model with no structuring of infectives with respect to parasite load (Dieckmann 2002). Interestingly, one can equally prove that for a constant $\mu$ variation of *V* does not allow any evolutionarily singular point, instead it again evolves towards the minimum plausible value of $\epsilon$ to maximise transmission.

*Linear trade-off* The situation changes for a linear trade-off function $V(\epsilon )=c\epsilon$ ($c>0$) with $h(\epsilon )=\epsilon$ in mortality rate: two evolutionarily singular strategies appear. This can be seen from the Pairwise Invasibility Plot (PIP) as shown in Fig. 2a. A PIP plot shows the sign of invasion fitness in the $(\epsilon _{r},\epsilon _{m})$ plane. In the figure, the grey regions represent the domain for which the mutant can successfully invade, whereas the white regions represent those regions for which the invasion of a rare mutant is not possible. Singular points in a PIP will be located at the intersection of these regions and the principal diagonal. For an ESS strategy, the sign of invasion fitness above and below the point (i.e. along the line passing through this point) should be negative (Geritz et al. 1998). Thus no mutants within this neighbourhood are able to successfully invade this singular point. As shown by (3.19), for the given model ESS-stability implies convergence stability hence this condition is sufficient in determining the stability of each singular point. Therefore in Fig. 2a it can be observed that the singular point at $\epsilon _{1}^{*}$ is ESS-stable and convergence stable (this can also be confirmed through the use of the second derivatives), whereas $\epsilon _{2}^{*}$ is not ESS-stable and therefore is not convergent stable. In other words, $\epsilon _{1}^{*}$ is an evolutionary attractor whereas $\epsilon _{2}^{*}$ is an evolutionary repellor. The long-term evolutionary outcome in the system will depend on initial conditions. If the initial parasite strain has $\epsilon <\epsilon _{2}^{*}$, the evolution will end up at an ESS $\epsilon _{1}^{*}$, whereas for $\epsilon >\epsilon _{2}^{*}$, the virulence and the transmission rate will be always growing until they reach their maximum biologically plausible values.

**Fig. 3:**
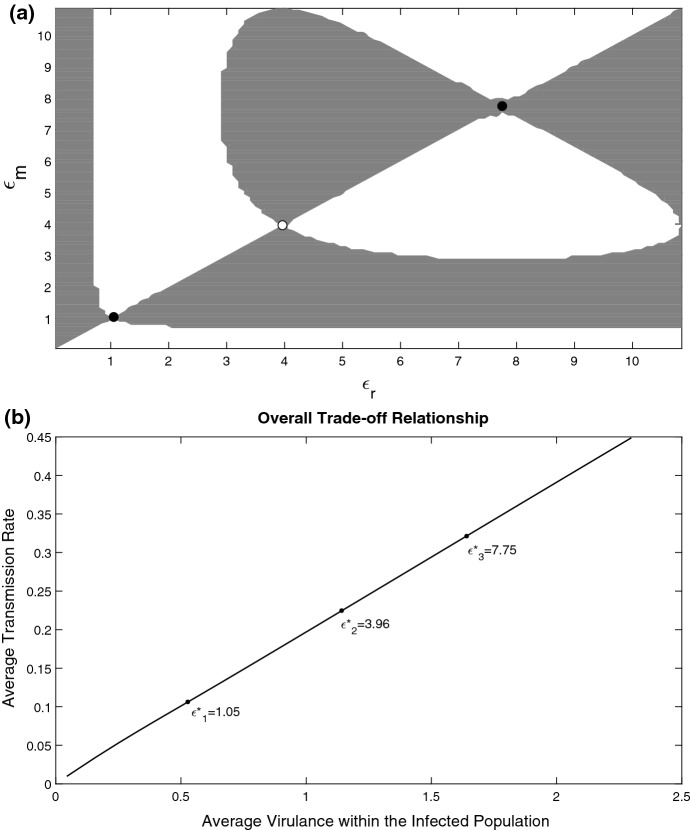
**a** Pairwise Invasibility Plot (PIP) describing long-term evolution in the system with $V(\epsilon )= \widetilde{c}\frac{\epsilon }{k+\epsilon }$ and $h(\epsilon )=\epsilon$ in $\mu (x,\epsilon )$. Here $g(\epsilon )\equiv 1$. Invasion fitness is plotted in the plane $(\epsilon _{r},\epsilon _{m})$ using (3.1). The grey regions represent a positive invasion fitness and the white regions represent a negative invasion fitness. The two evolutionary singular strategies are the evolutionary attractor shown by a black filled circle and an evolutionary repellor shown by the open circle. **b** Overall trade-off relationship between the average virulence and the average transmission rate across the parasite burden. The trade-off is computed by varying values of the evolutionary parameter $\epsilon$ within the viable infection range. The singular points are shown by the three black points and correspond to those presented in panel **a**. With ${\widetilde{c}}=800$, $k=1600$ and all other parameters are as in Fig. 2

**Fig. 4:**
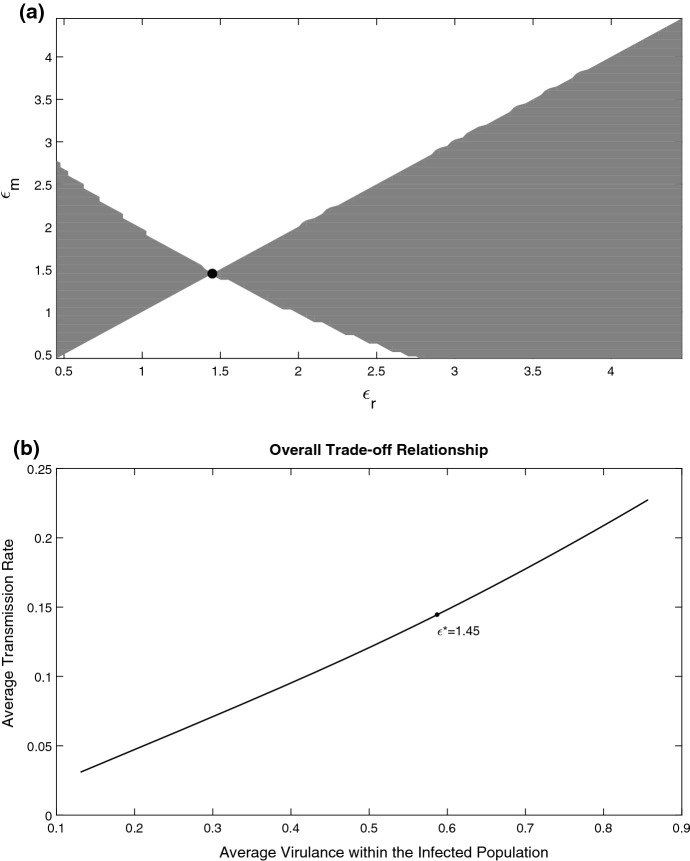
**a** Pairwise Invasibility Plot (PIP) describing long-term evolution in the system with $V(\epsilon )= const$, $\mu (x)$ and $\alpha (x,\epsilon )$ given by (2.4) with $g(\epsilon )=\epsilon$. Invasion fitness is plotted in the plane $(\epsilon _{r},\epsilon _{m})$ using (3.18). The grey regions represent a positive invasion fitness and the white regions represent a negative invasion fitness. The evolutionary singular strategy is an evolutionary attractor shown by a black filled circle. **b** Overall trade-off relationship between the average virulence and the average transmission rate across the parasite burden. The trade-off is computed by varying values of the evolutionary parameter $\epsilon$ within the viable infection range. The singular point is shown by the black point and corresponds to that of presented in panel **a** with $\mathrm{V}=0.6$ and all other parameters are as in Fig. 2

It is insightful to plot the overall trade-off relationship between the virulence understood as the total extra mortality rate due to parasites and the average transmission rate. Those values are computed at the stationary state of the system and shown in Fig. 2b. The figure also shows the two singular points obtained in the related PIP. The graph shows an almost linear global trade-off between virulence and transmission. Interestingly, a classical SI model with a linear trade-off between virulence and disease transmission predicts an ever-increasing virulence without any ESS state possible (Dieckmann 2002). Our simulation shows that the occurrence of an ESS is due to variation of the stationary distribution of parasite burden $i_{*}$ of the resident as a result of a change of $\epsilon$, which is clearly impossible in an unstructured model. The corresponding stationary distributions of parasite burden $i_{*}$ are presented in the supplementary material.

*Trade-off with saturation*

Following previous studies (Dieckmann 2002), we now add effects of saturation into the trade-off for $V(\epsilon )$ and consider the Monod parametrisation of the form $V(\epsilon )={\widetilde{c}}\frac{\epsilon }{k+\epsilon }$, where ${\widetilde{c}}$ and *k* are chosen such that *V* initially has the same slope as the linear trade-off. We find that the two previously found singular points in Fig. 2 still persist. However, saturation in $V(\epsilon )$ results in the appearance of another singular point. The corresponding PIP is shown in Fig. 3a. One can see that we now have evolutionary bi-stability, where two ESS strategies $\epsilon _{1}^{*} <\epsilon _{3}^{*}$ (which are also convergent stable) are separated by an evolutionary repellor $\epsilon _{2}^{*}$. The initial resident will evolve to $\epsilon _{1}^{*}$ if its initial $\epsilon$ is less than $\epsilon _{2}^{*}$ and will evolve to $\epsilon _{3}^{*}$ if it is larger than $\epsilon _{2}^{*}$. Thus depending on the starting evolutionary point, we will have either more virulent or a benign parasite strain. The ever-increasing $\epsilon$ does not occur anymore as in the example with the linear trade-off. The corresponding overall (average) trade-off plot is shown in Fig. 3b. This figure shows an almost linear dependence between the average virulence and transmission rate despite a non-linear function *V*(*D*), thus the emergence of ESS is due to alteration the stationary distribution of parasite load $i_{*}(x)$ shown in the supplementary material.

#### Trade-off between infection load growth $\alpha$ and transmission rate *V*

The trade-off between parasite virulence and transmission rate can actually have different underlying mechanisms. In this section, we consider the case where an increase in the parasite strength $\epsilon$ does not affect the mortality of the host $\mu$ but enhances the infection growth rate $\alpha$ and the transmission coefficient *V*. Mathematically, this can be described by (2.4). As before, we consider different parameterisations of $V(\epsilon )$.

*No trade-off* We start with the basic case of no trade-off, with *V* being a constant parameter being independent of the evolutionary parameter $\epsilon$. Unlike the previous case, the variation of a single growth rate $\alpha$ via changing $\epsilon$ would result in achieving an ESS in the system which is shown in the PIP in Fig. 4a. The ESS is shown by a black filled point. The corresponding overall trade-off between the virulence and the transmission rate is demonstrated in Fig. 4b. One can see that this dependence is non-linear. The important message from this illustrative example is that in the case of a structured population, variation of a single parameter (the parasite growth rate) would result in modification of the global traits like the overall virulence and the overall transmission rate due to alteration of the parasite burden distribution even if the parameters describing $\mu$ and $V(\epsilon )$ remain the same.

**Fig. 5:**
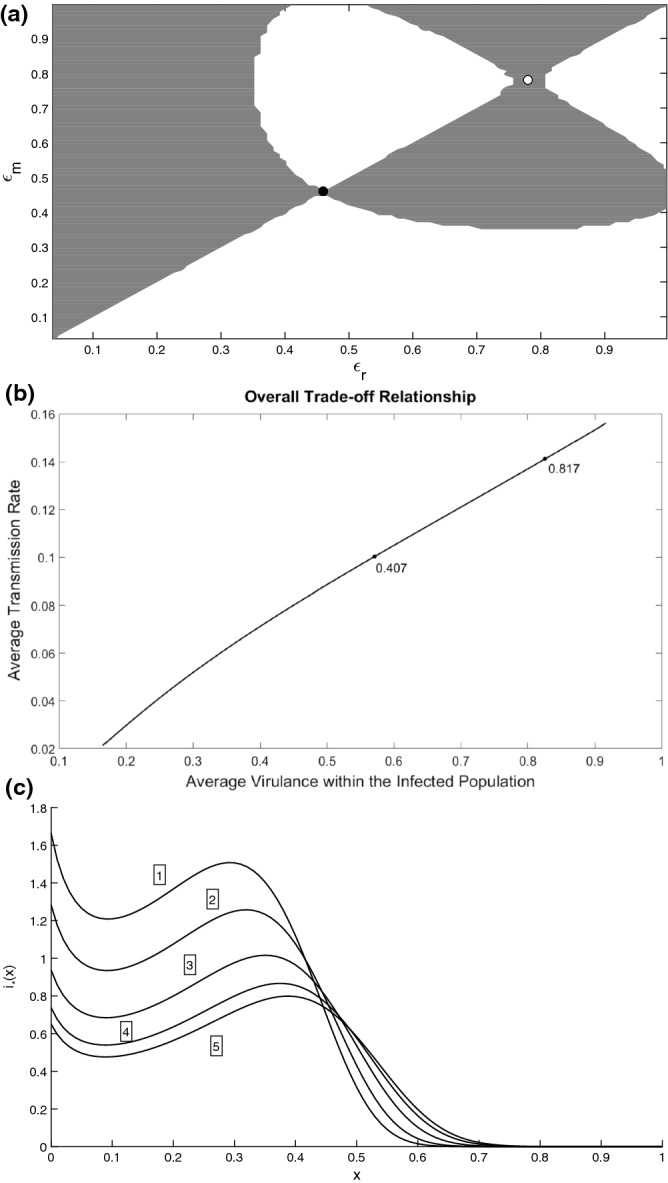
**a** Pairwise Invasibility Plot (PIP) describing long-term evolution in the system with $V(\epsilon )= \widetilde{c_1}\frac{\epsilon }{k_1+\epsilon }+\widetilde{c_2} \frac{\epsilon }{k_2+\epsilon }$ and $\alpha (x,\epsilon )$ given by (2.4) with $g(\epsilon )=\epsilon$. Invasion fitness is plotted in the plane $(\epsilon _{r},\epsilon _{m})$ using (3.18). The grey regions represent a positive invasion fitness and the white regions represent a negative invasion fitness. The two singular points are an evolutionary attractor shown by a black filled circle and an evolutionary repellor shown by an open circle. **b** Overall trade-off relationship between the average virulence and the average transmission rate across the parasite burden. The trade-off is computed by varying values of the evolutionary parameter $\epsilon$ within the viable infection range. The singular points are shown by the two black points and correspond to those found in panel **a**. **c** Parasite burden distributions for different parasite fitnesses $\epsilon$ at and around the singular points, the distributions are given for $\epsilon =0.56$, $\epsilon =\varepsilon ^*_1=0.46$, $\epsilon =0.62$, $\epsilon =\varepsilon ^*_2=0.78$, $\epsilon =0.88$. With $\widetilde{c_1}=10$, $k_1=75$, $\widetilde{c_2}=0.83$, $k_2=0.05$ and all other parameters are as in Fig. 2

*Linear trade-off and trade-off with saturation* We further consider scenarios where $V(\epsilon )$ is either a linear or a Monod function. We found that for the considered parameterisations of *V* the structure of PIP topologically remained the same (the graphs are not shown for brevity). We also considered a sum of two Monod functions with close parameters. In this case, an extra singular point appears in the diagram which is an evolutionary repellor. An example of such a situation is shown in the PIP in Fig. 5a. The corresponding overall trade-off is shown in Fig. 5b. Some examples of the parasite burden plots for different values of $\epsilon$ are shown in Fig. 5c. The figure demonstrates that the stationary profiles $i_*(x)$ gradually shift towards higher infection loads with an increase of the parasite fitness, thus the overall infection load increases with $\epsilon$ due to re-distribution of the parasite load.

#### Trade-off between infection load growth $\alpha$ and mortality $\mu$

**Fig. 6:**
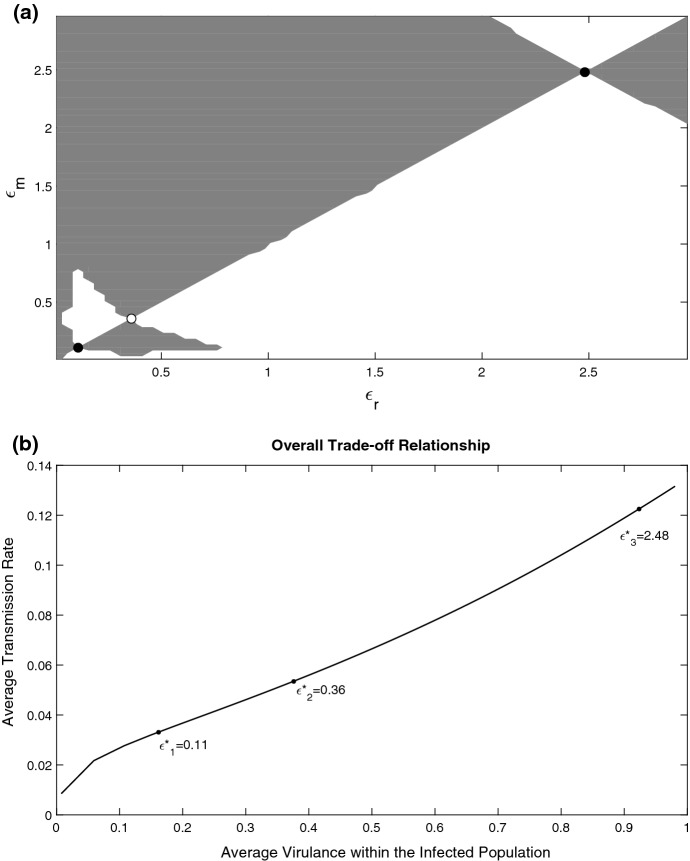
**a** Pairwise Invasibility Plot (PIP) describing long-term evolution in the system with evolving mortality and parasite growth rate $\mu (x,\epsilon )$ and $\alpha (x,\epsilon )$. Here $h(\epsilon )=0.2\frac{\epsilon }{\epsilon +0.1}$, $g(\epsilon )=\epsilon$, $V=0.6$. Invasion fitness is plotted in the plane $(\epsilon _{r},\epsilon _{m})$ using (3.18). The grey regions represent a positive invasion fitness and the white regions represent a negative invasion fitness. The two singular points are an evolutionary attractor shown by a black filled circle and an evolutionary repellor shown by an open circle. **b** Overall trade-off relationship between the average virulence and the average transmission rate across the parasite burden. The trade-off is computed by varying values of the evolutionary parameter $\epsilon$ within the viable infection range. The singular points are shown by the two black points and correspond to those found in panel **a**. Other parameters are as in Fig. 2

Finally, we explore the case where the parasite strength $\epsilon$ affects the parasite growth rate and the extra mortality due to infection. Mathematically we consider the three following cases for the trade-off functions *h* and *g* including linear an saturate dependencies: (i) $g(\epsilon )=\epsilon$ and $h(\epsilon )=\epsilon$; (ii) $g(\epsilon )=c\epsilon /(k+\epsilon )$ and $h(\epsilon )=\epsilon$; (iii) $g(\epsilon )=\epsilon$ and $h(\epsilon )=c\epsilon /(k+\epsilon )$. We obtained the following results. In cases (i) and (ii) we have a single ESS (the corresponding diagrams are not shown here for brevity). In case (iii), we have a bi-stability of ESSs presented in Fig. 6a: the middle singular point is an evolutionary repellor and the other two are evolutionary attractors. The corresponding overall trade-off plot can be seen in Fig. 6b. Interestingly, the global trade-off shows an inflection point with a further acceleration even if the dependencies on $\epsilon$ in (2.4) and (2.5) are described by a linear or a sub-linear function.

## Discussion and conclusions

In this paper, we extended previous studies on modelling evolution of virulence to the realistic case where the infection burden (i.e. the amount of parasite per host) is highly variable from individual to individual. Our work is motivated by a number of empirical studies reporting large variability of parasite load within infected host and the fact that mortality and the transmission rate would strongly depend on the number of parasites per host (Hudson et al. 1992; Temple 1987; Craig et al. 2006; Klimpel et al. 2006). Mathematically, we analyse an SI-type model with continuous structuring in terms of parasite load. Note that despite the fact that population structuring was incorporated in a number of the previous studies in mathematical epidemiology, most of previous models studied previously introduced variability of host individuals with respect to age or body size (Li and Brauer 2008; Martcheva 2015). The evolution of virulence in the current work was modelled based on the adaptive dynamics paradigm considering separation of evolutionary and ecological timescales. Note that our main analytical results (e.g. stability analysis, the expression for invasion fitness, types of evolutionary singular points) are valid for arbitrary trade-offs between parameters $\alpha$, $\mu$, $\rho$, ${\varLambda }$.

Our study demonstrates that principles of adaptive dynamics can be readily extended to the case of continuous structuring with respect to infection load: the major difference being that in our case invasion fitness is determined by a more complex equation (3.18). A major mathematical challenge, however, is to prove analytically the stability of the positive stationary state of the system (to use Eq. (3.18) we need to make sure that the resident population is constant), which cannot be easily done unlike in the case of the classic unstructured SI model. Nevertheless, using analytical expression for the invasion fitness we proved that for arbitrary trade-offs between parameters in both structured and unstructured models the possible evolutionary endpoints are ESSs, thus branching behaviour is not possible. Note that the restriction on branching in model (2.1) is due to the fact that the mortality rate of the infected subpopulation *i* is density independent. The absence of branching is probably not that surprising if one recalls that the equation for the mutant strain contains a single environmental feedback, the density $S_*$ which was shown to restrict branching behaviour (Gyllenberg and Service 2011; Lion and Metz 2018). We predict that branching in (2.1) might be possible via adding an additional environmental feedback into the equation for the infected subpopulation, for example, virulence-dependent predation, as it was shown for an unstructured model in Morozov and Best (2012). Actually, finding branching in more complicated models in distributed infection load would be a natural extension of the current research.

As illustrative and biologically meaningful examples, we considered evolutionary outcomes for various pairwise connections between the growth rate, mortality and transmission rate. We found evolutionary bistability for various trade-off scenarios: the eventual outcome—benign or virulent strain—will depend on the initial parasite strain. Another interesting result is construction of overall trade-off functions, which we can measure using empirical data (e.g. Alizon et al. 2009). In particular, this enhances possible classes of functions that we can potentially use in unstructured models and reinforces the critical functions analysis approach which admits a large class of trade-off dependencies (Geritz et al. 2007). On the other hand, the model with distributed parasite load has a structural particularity: the explicit function $\alpha (x,\epsilon )$ describing the growth of parasite inside the host. Such a function is clearly absent in the unstructured model. We argue that the explicit modelling of the growth of parasites load will have some advantages. For example, the variation of a single evolutionary parameter in $\alpha (x,\epsilon )$—and keeping the other model parameters constant—may result in achieving an intermediate evolutionary stable virulence, which would not be possible in the corresponding unstructured model (Dieckmann 2002). The explanation for that is that an increase in the growth rate of parasite results in higher values of infection load *x* which translates itself into a higher mortality and thus results in a higher disease transmission. Thus, the trade-off between overall virulence and the transmission rate becomes an emerging property of the host-parasite system.

**Fig. 7:**
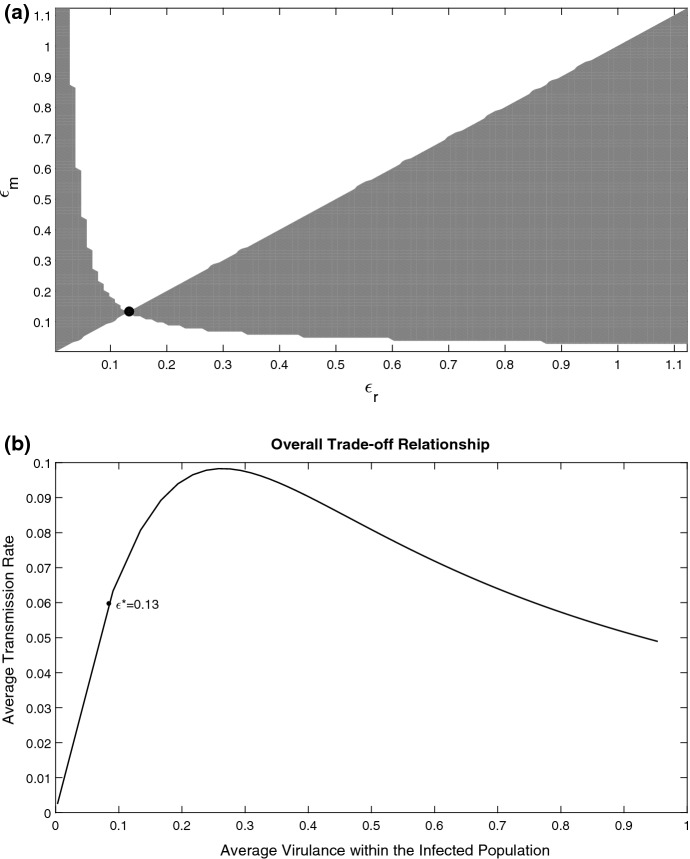
**a** Pairwise Invasibility Plot (PIP) describing long-term evolution in the system with $V(\epsilon )= {\widetilde{c}}\frac{\epsilon }{k+\epsilon }$ and $\mu (x,\epsilon )$ given by (2.5). The parasite growth rate is given by $\alpha (x)=C_0-x$ with $C_0=1.01$. The grey regions represent a positive invasion fitness and the white regions represent a negative invasion fitness. The evolutionary stable strategy is shown by a backfilled circle. **b** Overall trade-off relationship between the average virulence and the average transmission rate across the parasite burden. The trade-off is computed by varying values of the evolutionary parameter $\epsilon$ within the viable infection range. The singular point is shown by the black point and corresponds to this presented in panel **a**. With ${\widetilde{c}}=1$, $k=2$ and all other parameters are as in Fig. 2

We found that evolutionary outcomes in the system will depend on the mathematical parametrisation of the parasite growth rate $\alpha (x,\epsilon )$. In most simulations we considered the unimodal function (2.4). However, we also considered some other options (e.g. the logistic function, a constant $\alpha$ or even monotonically decreasing growth rate). An interesting behaviour is observed in the case where the growth rate is a linearly decreasing function of infection load $\alpha (x)=C_0-x$ with $C_0=1.01$ (here $\alpha$ is independent of $\epsilon$) and we considered the Monod trade-off between *V* and $\mu$. Biologically, such a scenario can occur in the situation where the response of the immune system of the host becomes stronger with an increase in parasite load. The corresponding PIP can be seen in Fig. 7a. Surprisingly, the shape of the overall trade-off between the transmission and virulence has an intermediate maximum in Fig. 7b. For high values of virulence, the transmission rate reduces. The explanation of this unusual behaviour comes from the corresponding stationary profiles $i_*(x)$ (see Supplementary Material): the distribution of infected gradually moves towards lower infection loads as the virulence increases.

Finally, we should stress that a major challenge of modelling evolution in systems with distributed parasite loads is an apparent increase in the system complexity: in such systems, one needs to parameterise extra functions $\alpha$, $\mu$, $\rho$, ${\varLambda }$ and different parameterisations may provide fully distinct results. Moreover, the choice of an evolutionary parameter and trade-offs may strongly affect the evolutionary outcomes. We should say, however, that this drawback can be partially compensated by the opportunity of revealing $\alpha (x,\epsilon )$, $\mu (x,\epsilon )$, $\rho (x,\epsilon )$, ${\varLambda }(x,\epsilon )$ directly from data (e.g. Fig. 1): this can be done on short (ecological) time scales. Note that in many ecological cases the recovery is absent which simplifies the task. On the other hand, we believe the current model—which combines both phenomenological and first principle approaches—can be extended using a more mechanistic approach of describing within host dynamics of parasite (e.g. based on first principles) and this will be an interesting subject for future studies. For example, one can consider as evolutionary parameters the ones which describe parasite reproduction, growth and survival inside the host. Then we can explore possible mechanistic (biological) connection of those parameters to host mortality and transmission rates. The overall trade-off between virulence and transmission at the scale of the population will emerge as the result of interplay between evolution of ‘microparameters’ and the corresponding alteration of the shape of frequency distribution of infection load. This promising approach of mechanistically revealing trade-offs—which emerge as a property of the system and are not defined by some prescribed functions—is currently gaining support in the literature (Sieber and Gudelj 2014; Meyer et al. 2015). Finally, another important direction (not considered here) is assuming the possibility of co-existence of different strains within a single host.

### Electronic supplementary material

Below is the link to the electronic supplementary material.

Supplementary material 1 (pdf 559 KB)

## Appendix A

Here we prove Proposition 4 providing sufficient conditions of stability of the positive stationary state of the model.

### Proof

We introduce the following linear operators

4.1 $$\begin{aligned} \begin{aligned} {\mathcal {A}}\begin{pmatrix} j \\ U \end{pmatrix}&= \begin{pmatrix} -\alpha (\cdot ,\epsilon )j_x \\ \left( r(S_*)+r'(S_*)S_*-f'(S_*)V(\epsilon )\int _0^1i_*(x){\varLambda }(x)\,\mathrm {d}x\right) U\\ \end{pmatrix}, \\ \text {D}({\mathcal {A}})&= \left\{ \begin{pmatrix} j \\ U \\ \end{pmatrix}\in W^{1,1}(0,1)\times {\mathbb {R}} \, \vert \, \alpha (0,\epsilon )j(0)={\varPhi }\begin{pmatrix} j \\ U \\ \end{pmatrix}\right\} , \\ {\varPhi }\begin{pmatrix} j \\ U \end{pmatrix}&= f(S_*)V(\epsilon )\int _0^1{\varLambda }(x)j(x)\,\mathrm {d}x+U\,f'(S_*)V(\epsilon )\int _0^1{\varLambda }(x)i_*(x)\,\mathrm {d}x, \\ \text {D}({\varPhi })&={\mathcal {X}} \\ {\mathcal {B}}\begin{pmatrix} j \\ U \end{pmatrix}&= \begin{pmatrix} -\left( \alpha _x(\cdot ,\epsilon ) +\rho (\cdot ,\epsilon )+\mu (\cdot ,\epsilon )\right) j\\ \int _0^1\left( \rho (x,\epsilon )-f(S_*)V(\epsilon ){\varLambda }(x)\right) j(x)\,\mathrm {d}x \\ \end{pmatrix},\quad \text {D}({\mathcal {B}})={\mathcal {X}}, \end{aligned} \end{aligned}$$

and recast the linearised problem (3.7) as an abstract Cauchy problem on ${\mathcal {X}}$ as

4.2 $$\begin{aligned} \frac{\mathrm {d}}{\mathrm {d}t}\begin{pmatrix} j \\ U \\ \end{pmatrix} =({\mathcal {A}}+{\mathcal {B}})\begin{pmatrix} j \\ U \\ \end{pmatrix}, \quad \begin{pmatrix} j(0) \\ U(0) \\ \end{pmatrix}=\begin{pmatrix} j_0 \\ U_0 \\ \end{pmatrix}\in {\mathcal {X}}. \end{aligned}$$

We are going to show that there exists a $\kappa >0$ such that ${\mathcal {A}}+{\mathcal {B}}+\kappa {\mathcal {I}}$ is dissipative. Then the Lumer–Phillips theorem [see e.g. Theorem 3.15 in Engel and Nagel (2000, Ch. II)] implies that ${\mathcal {A}}+{\mathcal {B}}+\kappa {\mathcal {I}}$ generates a contraction semigroup on ${\mathcal {X}}$ (note that we have already established that ${\mathcal {A}}+{\mathcal {B}}$ generates a semigroup of bounded linear operators on ${\mathcal {X}}$, and therefore so does ${\mathcal {A}}+{\mathcal {B}}+\kappa {\mathcal {I}}$); and in particular the semigroup $\left\{ {\mathcal {T}}(t)\right\} _{t\ge 0}$ generated by ${\mathcal {A}}+{\mathcal {B}}$ satisfies $||{\mathcal {T}}(t)||_{\mathcal {X}}\le e^{-\kappa \,t}, \quad \forall \,t\ge 0$. We note that a similar dissipativity approach was employed for simpler models in Farkas and Hagen (2009, 2010), Farkas and Hinow (2010).

Recall for example from (Engel and Nagel 2000, Ch. II) that a linear operator ${\mathcal {L}}$ with domain D(${\mathcal {L}}$) on a Banach space ${\mathcal {Y}}$ is called dissipative if

4.3 $$\begin{aligned} ||(\lambda \,{\mathcal {I}}-{\mathcal {L}})\,y||\ge \lambda ||y||,\quad \forall \,\lambda >0,\,\,\forall \,y\in \text {D}({\mathcal {L}}). \end{aligned}$$

To verify that indeed for some $\kappa >0$ the linear operator ${\mathcal {A}}+{\mathcal {B}}+\kappa {\mathcal {I}}$ is dissipative, consider the equation

4.4 $$\begin{aligned} \begin{pmatrix} v \\ V \\ \end{pmatrix}= \lambda \left( {\mathcal {A}}+{\mathcal {B}}+\kappa {\mathcal {I}}\right) \begin{pmatrix} v \\ V \\ \end{pmatrix}+ \begin{pmatrix} h \\ H \\ \end{pmatrix}, \end{aligned}$$

where $$\begin{pmatrix} h \\ H \\ \end{pmatrix}\in {\mathcal {X}}$$, $$\lambda >0$$, and $$\begin{pmatrix} v \\ V \\ \end{pmatrix}\in \text {D}\left( {\mathcal {A}}+{\mathcal {B}}+ \kappa {\mathcal {I}}\right) =\text {D}({\mathcal {A}})$$.

From (4.4) we obtain the following estimates (below ‘sgn’ stands for the signum function)

4.5 $$\begin{aligned} \begin{aligned} \left\| \begin{pmatrix} v \\ V \\ \end{pmatrix}\right\| _{\mathcal {X}} =&\int _0^1 \vert v(x)\vert \,\mathrm {d}x+|V| = \int _0^1 v(x)\,\text {sgn}\,v(x)\,\mathrm {d}x+V\,\text {sgn}\,V \\ =&\int _0^1 h(x)\,\text {sgn}\,v(x)\,\mathrm {d}x + H\,\text {sgn}\,V -\lambda \int _0^1\left( \alpha (x,\epsilon )v(x)\right) _x\,\text {sgn}\, v(x)\,\mathrm {d}x \\&+\lambda \int _0^1(\kappa -\rho (x,\epsilon )-\mu (x,\epsilon ))\vert v(x)\vert \,\mathrm {d}x \\&+\lambda \,\text {sgn}\,V\int _0^1(\rho (x,\epsilon )- f(S_*)V(\epsilon ){\varLambda }(x))v(x)\,\mathrm {d}x \\&+\lambda \,\vert V\vert \left( r(S_*)+r'(S_*)S_*-f'(S_*)V(\epsilon )\int _0^1i_*(x){\varLambda }(x)\,\mathrm {d}x\right) +\lambda \,\kappa \,\vert V\vert \\ \le&\int _0^1 |h(x)|\,\mathrm {d}x +|H| +\lambda \,\alpha (0,\epsilon )\,|v(0)| \\&+\lambda \int _0^1(\kappa -\rho (x,\epsilon )-\mu (x,\epsilon ))\vert v(x)\vert \,\mathrm {d}x \\&+\lambda \,\text {sgn}\,V\int _0^1(\rho (x,\epsilon )-f(S_*)V(\epsilon ){\varLambda }(x))v(x)\,\mathrm {d}x \\&+\lambda \,\vert V\vert \left( r(S_*)+r'(S_*)S_*-f'(S_*)V(\epsilon )\int _0^1i_*(x){\varLambda }(x)\,\mathrm {d}x\right) +\lambda \,\kappa \,\vert V\vert \\ \le&\left\| \begin{pmatrix} h \\ H \\ \end{pmatrix} \right\| _{\mathcal {X}}+\lambda f(S_*)V(\epsilon )\int _0^1 {\varLambda }(x)|v(x)|\,\mathrm {d}x \\&+\lambda \left| Vf'(S_*)V(\epsilon )\int _0^1{\varLambda }(x)i_*(x)\,\mathrm {d}x\right| \\&-\lambda \int _0^1\rho (x,\epsilon )\vert v(x)\vert \,\mathrm {d}x+ \lambda \int _0^1\rho (x,\epsilon )\,\text {sgn}\,Vv(x)\,\mathrm {d}x \\&+\lambda \int _0^1(\kappa -\mu (x,\epsilon ))\vert v(x)\vert \, \mathrm {d}x-\lambda \,\text {sgn}\,V f(S_*)V(\epsilon )\int _0^1{\varLambda }(x)v(x)\,\mathrm {d}x \\&+\lambda \vert V\vert \left( r(S_*)+r'(S_*)S_*+\kappa \right) -\lambda \left| V \right| f'(S_*)V(\epsilon )\int _0^1{\varLambda }(x)i_*(x)\,\mathrm {d}x \\ \le&\left\| \begin{pmatrix} h \\ H \\ \end{pmatrix} \right\| _{\mathcal {X}} +\lambda f(S_*)V(\epsilon )\int _0^1 {\varLambda }(x)|v(x)|\,\mathrm {d}x \\&+\lambda \int _0^1(\kappa -\mu (x,\epsilon ))\vert v(x)\vert \, \mathrm {d}x-\lambda f(S_*)V(\epsilon )\int _0^1{\varLambda }(x)\,\text {sgn}\,Vv(x)\,\mathrm {d}x \\ \le&\left\| \begin{pmatrix} h \\ H \\ \end{pmatrix} \right\| _{\mathcal {X}} + \lambda \int _0^1\left( \kappa - \mu (x,\epsilon )+2f(S_*)V(\epsilon ){\varLambda }(x)\right) |v(x)|\,\mathrm {d}x \\ \le&\left\| \begin{pmatrix} h \\ H \\ \end{pmatrix}\right\| _{\mathcal {X}}, \end{aligned} \end{aligned}$$

for some $$\kappa>0,\,\,\forall \,\lambda >0,\,\,\forall \begin{pmatrix} v \\ V \\ \end{pmatrix}\in \text {D}( {\mathcal {A}})$$, if the conditions in (3.15) hold true.

To obtain the estimate $-\lambda \int _0^1 \left( \alpha (x, \epsilon )v(x)\right) _x\,\text {sgn}\, v(x)\,\mathrm {d}x\le \lambda \alpha (0,\epsilon )|v(0)|$ we divide the interval [0, 1] into the countable union of disjoint subintervals as follows:

$$\begin{aligned} (0,1)=\left( \cup _i (a_i,b_i)\right) \cup \left( \cup _{{\hat{i}}} (a_{{\hat{i}}},b_{{\hat{i}}})\right) \cup \left( \cup _j(a_j,b_j)\right) , \end{aligned}$$

such that $v>0$ on $(a_i,b_i)\,\forall \,i$, $v<0$ on $(a_{{\hat{i}}},b_{{\hat{i}}})\,\forall \,{\hat{i}}$, and $v=0$ on $(a_j,b_j)\,\forall \,j$. Then we have

$$\begin{aligned}&-\lambda \int _0^1(\alpha (x,\epsilon )v(x))_x\,\text {sgn}\, v(x)\,\mathrm {d}x \\ =&-\lambda \left( \sum _i\int _{a_i}^{b_i}(\alpha (x,\epsilon )v(x))_x\,\mathrm {d}x -\sum _{{\hat{i}}}\int _{a_{{\hat{i}}}}^{b_{{\hat{i}}}} (\alpha (x,\epsilon )v(x))_x\,\mathrm {d}x\right) \\ =&-\lambda \left( \sum _i(\alpha (b_i,\epsilon ) v(b_i)-\alpha (a_i,\epsilon )v(a_i))+\sum _{{\hat{i}}} (\alpha (a_{{\hat{i}}},\epsilon )v(a_{{\hat{i}}})-\alpha (b_{{\hat{i}}},\epsilon )v(b_{{\hat{i}}}))\right) \\ \le&\,\,\lambda \,\alpha (0,\epsilon )|v(0)|, \end{aligned}$$

since we have $v(a_i)=v(b_i)=v(a_{{\hat{i}}})=v(b_{{\hat{i}}})=0\,\,\forall \,i,{\hat{i}},$ unless $a_i=0$ or $a_{{\hat{i}}}=0$ and $b_i=1$ or $b_{{\hat{i}}}=0$.

The estimate (4.5) shows that ${\mathcal {A}}+{\mathcal {B}}+\kappa {\mathcal {I}}$ is indeed dissipative, and therefore the semigroup $\left\{ {\mathcal {T}}(t)\right\} _{t\ge 0}$ generated by ${\mathcal {A}}+{\mathcal {B}}$ is uniformly exponentially stable, which concludes the proof. $\square$

## Appendix B

We differentiate (3.18) with respect to $\epsilon _{m}$ and $\epsilon _{r}$ to obtain

$$\begin{aligned} \begin{aligned}&\int _{0}^{1}\frac{{\varLambda }(x)}{\alpha (x)}\exp \left( -\intop _{0}^{x}\frac{\lambda +\rho (y)+ \mu (y,\epsilon _m)}{\alpha (y)}\,dy\right) \\&\qquad \left( V'_{\epsilon _{m}}(\epsilon _m)-V(\epsilon _m) \int _{0}^{1}\frac{\lambda '_{\epsilon _{m}}+\mu '_{\epsilon _{m}} (y,\epsilon _m)}{\alpha (y)}\right) \,dx=0. \\&-V(\epsilon _m)\int _{0}^{1}\frac{{\varLambda }(x)}{\alpha (x)} \exp \left( -\intop _{0}^{x}\frac{\lambda +\rho (y)+\mu (y,\epsilon _m)}{\alpha (y)}\,dy\right) \int _{0}^{1}\frac{\lambda '_{\epsilon _{r}}}{\alpha (y)}\,dx \\&\quad = \int _{0}^{1}\frac{{\varLambda }(x)}{\alpha (x)}\exp \left( -\intop _{0}^{x}\frac{\rho (y)+\mu (y,\epsilon _r)}{\alpha (y)}\,dy\right) \left( V'_{\epsilon _{r}}(\epsilon _r) -V(\epsilon _r)\int _{0}^{1}\frac{\mu '_{\epsilon _{r}}(y,\epsilon _r)}{\alpha (y)}\right) \,dx. \end{aligned} \end{aligned}$$

We next differentiate the above expressions and evaluate them at the ESS to obtain

$$\begin{aligned} \begin{aligned}&\frac{\partial ^{2}\lambda (\epsilon _{r},\epsilon _{m})}{\partial \epsilon _{m}^{2}} \\&\quad =\frac{\displaystyle \int _{0}^{1}\frac{{\varLambda }(x)}{\alpha (x)} \exp \left( -\intop _{0}^{x}\frac{\rho (y)+\mu (y,\epsilon ^{*})}{\alpha (y)}\,dy\right) \left[ V''_{\epsilon ^{m}}(\epsilon ^{*})- 2V'_{\epsilon ^{m}}(\epsilon ^{*})\displaystyle \int _{0}^{x}\frac{\mu '_{\epsilon ^{m}} (y,\epsilon ^{*})}{\alpha (y)}\,dy\right] \,dx}{\displaystyle \int _{0}^{1} \frac{{\varLambda }(x)}{\alpha (x)}\exp \left( -\intop _{0}^{x} \frac{\rho (y)+\mu (y,\epsilon ^{*})}{\alpha (y)}\,dy\right) V(\epsilon ^{*})\displaystyle \int _{0}^{x}\frac{1}{\alpha (y)}\,dy\,dx}\\&\qquad +\frac{\displaystyle \int _{0}^{1}\frac{{\varLambda }(x)}{\alpha (x)} \exp \left( -\intop _{0}^{x}\frac{\rho (y)+\mu (y,\epsilon ^{x})}{\alpha (y)}\,dy\right) \left[ V(\epsilon ^{*})\left( \left( \displaystyle \int _{0}^{x}\frac{\mu '_{\epsilon ^{m}}(y, \epsilon ^{*})}{\alpha (y)}\,dy\right) ^{2}-\displaystyle \int _{0}^{x} \frac{\mu ''_{\epsilon ^{m}}(y,\epsilon ^{*})}{\alpha (y)} \,dy\right) \right] \,dx}{\displaystyle \int _{0}^{1}\frac{{\varLambda }(x)}{\alpha (x)}\exp \left( -\intop _{0}^{x}\frac{\rho (y)+ \mu (y,\epsilon ^{*})}{\alpha (y)}\,dy\right) V(\epsilon ^{*}) \displaystyle \int _{0}^{x}\frac{1}{\alpha (y)}\,dy\,dx}. \end{aligned} \end{aligned}$$

Similarly differentiating (3.18) twice with respect to $\epsilon _{r}$ and evaluated at the ESS $\epsilon ^{*}$ the following expression is obtained;

$$\begin{aligned} \begin{aligned}&V(\epsilon ^{*})\int _{0}^{1}\frac{{\varLambda }(x)}{\alpha (x)}\exp \left( -\intop _{0}^{x}\frac{\rho (y)+\mu (y,\epsilon ^{*})}{\alpha (y)}\,dy\right) \int _{0}^{x}\frac{-\frac{\partial ^{2} \lambda (\epsilon _{r},\epsilon _{m})}{\partial \epsilon _{r}^{2}}}{\alpha (y)}\,dy\,dx \\&\quad =\int _{0}^{1}\frac{{\varLambda }(x)}{\alpha (x)}\exp \left( -\intop _{0}^{x} \frac{\rho (y)+\mu (y,\epsilon ^{*})}{\alpha (y)}\,dy\right) \\&\qquad \left[ V''_{\epsilon ^{r}}(\epsilon ^{*})-2V'_{\epsilon ^{r}} (\epsilon ^{*})\int _{0}^{x}\frac{\mu '_{\epsilon ^{r}}(y, \epsilon ^{*})}{\alpha (y)}\,dy\right] \,dx\\&\qquad +\int _{0}^{1}\frac{{\varLambda }(x)}{\alpha (x)}\exp \left( -\intop _{0}^{x} \frac{\rho (y)+\mu (y,\epsilon ^{*})}{\alpha (y)}\,dy\right) \\&\qquad \left[ V(\epsilon ^{*})\left( \left( \int _{0}^{x}\frac{\mu '_{\epsilon ^{r}} (y,\epsilon ^{*})}{\alpha (y)}\,dy\right) ^{2}-\int _{0}^{x} \frac{\mu ''_{\epsilon ^{r}}(y,\epsilon ^{*})}{\alpha (y)}\,dy \right) \right] \,dx. \end{aligned} \end{aligned}$$

This expression can be simplified to

$$\begin{aligned} \begin{aligned}&-\frac{\partial ^{2}\lambda (\epsilon _{r},\epsilon _{m})}{\partial \epsilon _{m}^{2}}\\&\quad =\frac{\displaystyle \int _{0}^{1}\frac{{\varLambda }(x)}{\alpha (x)} \exp \left( -\intop _{0}^{x}\frac{\rho (y)+\mu (y,\epsilon ^{*})}{\alpha (y)} \,dy\right) \left[ V''_{\epsilon ^{r}}(\epsilon ^{*})-2V'_{\epsilon ^{r}} (\epsilon ^{*})\displaystyle \int _{0}^{x}\frac{\mu '_{\epsilon ^{r}}(y,\epsilon ^{*})}{\alpha (y)}\,dy\right] \,dx}{\displaystyle \int _{0}^{1}\frac{{\varLambda }(x)}{\alpha (x)} \exp \left( -\intop _{0}^{x}\frac{\rho (y)+\mu (y,\epsilon ^{*})}{\alpha (y)} \,dy\right) V(\epsilon ^{*})\displaystyle \int _{0}^{x}\frac{1}{\alpha (y)}\,dy\,dx}\\&\qquad +\frac{\displaystyle \int _{0}^{1}\frac{{\varLambda }(x)}{\alpha (x)}\exp \left( -\intop _{0}^{x} \frac{\rho (y)+\mu (y,\epsilon ^{x})}{\alpha (y)}\,dy\right) \left[ V(\epsilon ^{*})\left( \left( \displaystyle \int _{0}^{x}\frac{\mu '_{\epsilon ^{r}} (y,\epsilon ^{*})}{\alpha (y)}\,dy\right) ^{2}-\displaystyle \int _{0}^{x} \frac{\mu ''_{\epsilon ^{r}}(y,\epsilon ^{*})}{\alpha (y)}\,dy\right) \right] \,dx}{\displaystyle \int _{0}^{1}\frac{{\varLambda }(x)}{\alpha (x)}\exp \left( -\intop _{0}^{x}\frac{\rho (y)+\mu (y,\epsilon ^{*})}{\alpha (y)} \,dy\right) V(\epsilon ^{*})\displaystyle \int _{0}^{x}\frac{1}{\alpha (y)}\,dy\,dx}. \end{aligned} \end{aligned}$$
