# Supplementary material for: Modelling evolution of virulence in populations with a distributed parasite load
Source: J Math Biol. 2019 Apr 10;80(1):111–41. doi: 10.1007/s00285-019-01351-6 (PMC7012800; doi:10.1007/s00285-019-01351-6)
Supplement: Supplementary file 1 — Supplementary material 1 (pdf 559 KB) [file 285_2019_1351_MOESM1_ESM.pdf]

Supplementary Material for the paper 'Modelling Evolution of Virulence in Populations with a Distributed Parasite Load' by Sandhu et al.

Model Parameter Plots

Here we present the plot of the growth of parasite load defined as an increasing function of ϵ. With the assumptions that the initial growth rate at the minimal parasite load is positive and if the parasite strength vanishes, its growth is not possible anymore. We consider that

$$\alpha(x, \epsilon) = g(\epsilon)((x - B_1)^2(x + A_1)\exp(-D_1(x + A_1)) + C_1), \tag{0.1}$$

where A_1, B_1, C_1 and D_1 are positive parameters. We choose A_1 close to 0 and B_1 close to 1. Here $g(\epsilon)$ is a function describing the link between the parasite strength and the growth rate.

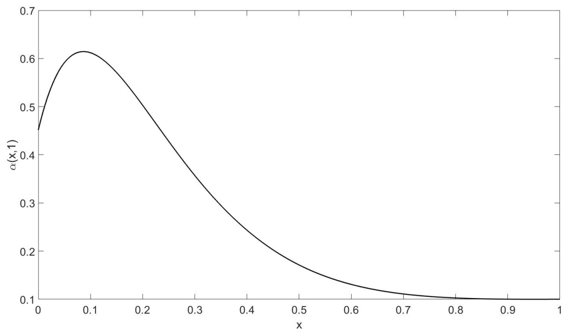

Fig. 1 Plot of the growth of parasite load at $\epsilon = 1$, with parameters given as $B_1 = 0.95$, $A_1 = 0.05$, $C_1 = 0.1$, $D_1 = 5$, the function $g(\epsilon)$ is given by $g(\epsilon) = 10\epsilon$.

Proof of Proposition 1

Proposition 1 *A parasite strain ϵ can successfully spread in a fully susceptible population at $\widetilde{S_*}$ provided that the basic reproduction number*

$$R_0(\epsilon) = f(\widetilde{S_*})V(\epsilon)\int_0^1 \exp\left(-\int_0^x \frac{\rho(y,\epsilon) + \mu(y,\epsilon)}{\alpha(y,\epsilon)}\,dy\right)\frac{\Lambda(x)}{\alpha(x,\epsilon)}\,dx \tag{0.2}$$

is greater than one. Otherwise, the parasite will go extinct. Therefore, the range of viable ϵ values to be considered further to determine possible evolutionary outcomes is determined by the condition $R_0(\epsilon) > 1$.

Suppose at the initial condition $(0, \widetilde{S_*})$ we introduce some infection, this population can be described by the following linear model.

$$
\begin{aligned}
i_t(x,t) + (\alpha(x,\epsilon) i(x,t))_x &= -\rho(x,\epsilon) i(x,t) - \mu(x,\epsilon) i(x,t), \\
\alpha(0,\epsilon) i(0,t) &= f(\widetilde{S_*}) \int_0^1 i(x,t) \varLambda(x) V(\epsilon)\, dx,
\end{aligned} \tag{0.3}
$$

To determine this leading eigenvalue we look for solutions of (0.3) in the form of a separable solution.

$$
i(x,t) = \exp\left(\lambda(\epsilon)\, t\right) \phi(x),
$$

Substitution of this solution into the model we obtain the following

$$
\lambda(\epsilon)\phi(x) + (\alpha(x,\epsilon)\phi(x))_x = -\rho(x,\epsilon)\phi(x) - \mu(x,\epsilon)\phi(x) \tag{0.4}
$$

This can be solved to give

$$
\frac{\alpha(x,\epsilon)\phi(x)}{\alpha(0,\epsilon)\phi(0)} = \exp\left(-\int_0^x \frac{\rho(y,\epsilon) + \mu(y,\epsilon) + \lambda(\epsilon)}{\alpha(y,\epsilon)}\, dy \right) \tag{0.5}
$$

By the boundary condition we have that

$$
1 = \frac{\alpha(0,\epsilon)\phi(0)}{\alpha(0,\epsilon)\phi(0)} = \frac{f(\widetilde{S_*})}{\alpha(0,\epsilon)\phi(0)} \int_0^1 \phi(x) \varLambda(x) V(\epsilon)\, dx \tag{0.6}
$$

substituting in our expression for $\phi(x)$ we have the following

$$
1 = f(\widetilde{S_*}) \int_0^1 \frac{1}{\alpha(x,\epsilon)} \exp\left(-\int_0^x \frac{\rho(y,\epsilon) + \mu(y,\epsilon) + \lambda(\epsilon)}{\alpha(y,\epsilon)}\, dy \right) \varLambda(x) V(\epsilon)\, dx \tag{0.7}
$$

Therefore if

$$
R_0(\epsilon) = f(\widetilde{S_*}) V(\epsilon) \int_0^1 \exp\left(-\int_0^x \frac{\rho(y,\epsilon) + \mu(y,\epsilon)}{\alpha(y,\epsilon)}\, dy \right) \frac{\varLambda(x)}{\alpha(x,\epsilon)}\, dx \tag{0.8}
$$

then we have the following relationship between λ and R_0

$$
R_0 > 0 \iff \lambda > 0
$$

$$
R_0 = 0 \iff \lambda = 0
$$

$$
R_0 < 0 \iff \lambda < 0
$$

Parasite Burden Plots

Here we present the graphs for stationary distributions of infected subpopulation $i_*(x)$ corresponding to the results presented in figures 2-7 in the main text of the paper. The details on model parameters and trade-off functions can be found in the figure captions in the main text.

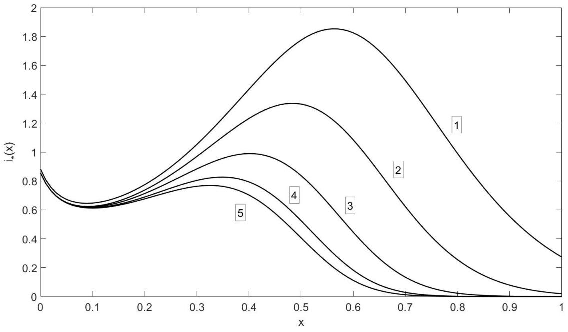

Fig. 2 Different infection distributions corresponding to Fig. 2 of the main text where we considered the linear trade-off between mortality μ and transmission rate V. The distributions are given for $\boxed{1}$ $\epsilon = 0.5$, $\boxed{2}$ $\epsilon = \epsilon*_1 = 1.00$, $\boxed{3}$ $\epsilon = 0.91$, $\boxed{4}$ $\epsilon = \epsilon*_2 = 2.82$, $\boxed{5}$ $\epsilon = 3.32$.

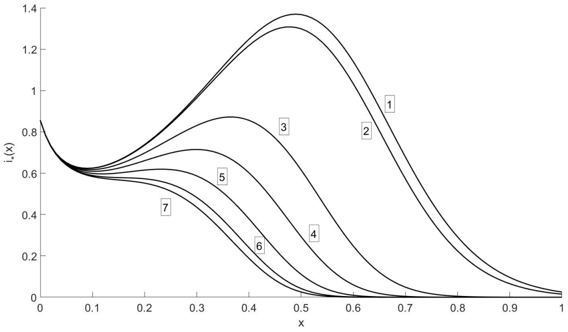

Fig. 3 Different infection distributions corresponding to Fig. 3 of the main text where the hyperbolic trade-off between mortality μ and transmission rate V was investigated. The distributions are given for $\boxed{1}$ $\epsilon = 0.95$, $\boxed{2}$ $\epsilon = \epsilon*_1 = 1.05$, $\boxed{3}$ $\epsilon = 2.51$, $\boxed{4}$ $\epsilon = \epsilon*_2 = 3.96$, $\boxed{5}$ $\epsilon = 5.86$, $\boxed{6}$ $\epsilon = \epsilon*_3 = 7.75$, $\boxed{7}$ $\epsilon = 8.75$.

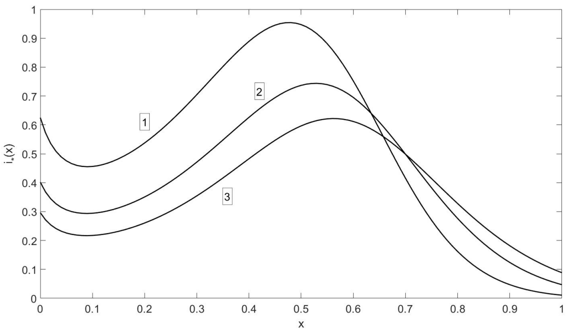

Fig. 4 Different infection distributions corresponding to Fig. 4 of the main text corresponding to the trade-off between infection load growth α and transmission rate V for the special case when V is some constant parameter. The distributions are given for $\boxed{1}$ $\epsilon = 0.95$, $\boxed{2}$ $\epsilon = \epsilon*_1 = 1.45$, $\boxed{3}$ $\epsilon = 1.95$.

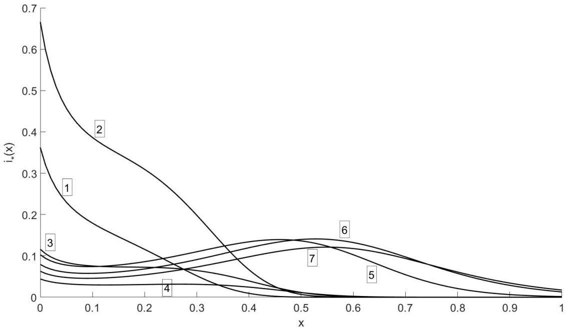

Fig. 5 Different infection distributions corresponding to Fig. 6 of the main text where we consider the trade-off between infection load growth α and mortality μ. The distributions are given for $\boxed{1}$ $\epsilon = 0.06$, $\boxed{2}$ $\epsilon = \epsilon*_1 = 0.11$, $\boxed{3}$ $\epsilon = 0.16$.

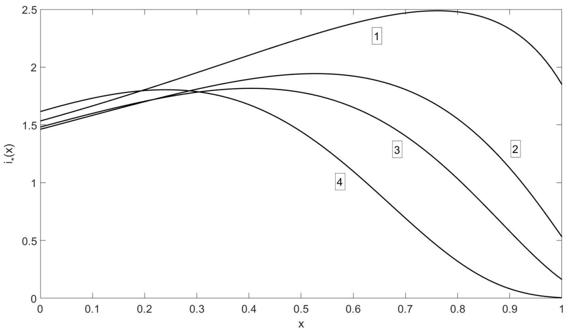

Fig. 6 Different infection distributions corresponding to Fig. 7 of the main text for the case where the growth rate is a linearly decreasing function of infection load $\alpha(x) = C_0 - x$ with $C_0 = 1.01$ and we consider the Monod trade-off between V and μ. The distributions are given for $\boxed{1}$ $\epsilon = 0.08$, $\boxed{2}$ $\epsilon = \epsilon{*}_1 = 0.13$, $\boxed{3}$ $\epsilon = 0.18$, $\boxed{4}$ $\epsilon = \epsilon{*}_2 = 0.33$.
